# Chromatoid Body Protein TDRD6 Supports Long 3’ UTR Triggered Nonsense Mediated mRNA Decay

**DOI:** 10.1371/journal.pgen.1005857

**Published:** 2016-05-05

**Authors:** Grigorios Fanourgakis, Mathias Lesche, Müge Akpinar, Andreas Dahl, Rolf Jessberger

**Affiliations:** 1 Institute of Physiological Chemistry, Medical Faculty Carl Gustav Carus, Technische Universität Dresden, Dresden, Germany; 2 Deep Sequencing Group SFB 655, Biotechnology Center, Technische Universität Dresden, Dresden, Germany; Cornell University, UNITED STATES

## Abstract

Chromatoid bodies (CBs) are spermiogenesis-specific organelles of largely unknown function. CBs harbor various RNA species, RNA-associated proteins and proteins of the tudor domain family like TDRD6, which is required for a proper CB architecture. Proteome analysis of purified CBs revealed components of the nonsense-mediated mRNA decay (NMD) machinery including UPF1. TDRD6 is essential for UPF1 localization to CBs, for UPF1-UPF2 and UPF1-MVH interactions. Upon removal of TDRD6, the association of several mRNAs with UPF1 and UPF2 is disturbed, and the long 3’ UTR-stimulated but not the downstream exon-exon junction triggered pathway of NMD is impaired. Reduced association of the long 3’ UTR mRNAs with UPF1 and UPF2 correlates with increased stability and enhanced translational activity. Thus, we identified TDRD6 within CBs as required for mRNA degradation, specifically the extended 3’ UTR-triggered NMD pathway, and provide evidence for the requirement of NMD in spermiogenesis. This function depends on TDRD6-promoted assembly of mRNA and decay enzymes in CBs.

## Author Summary

Tudor-domain containing protein 6 (TDRD6) is a central component of the chromatoid body (CB) in male germ cells. Chromatoid bodies, which are present in spermatids, contain RNA and protein, are not enclosed by membranes, and typically reside close to the nucleus. Without TDRD6, a much distorted CB structure is observed, and this work asked for the functional contribution of TDRD6 to spermatids. We found that TDRD6 is required for localization of an RNA degradation machinery to the CB. This so-called nonsense mediated decay (NMD) machinery, known from somatic cells, destroys mRNAs that feature premature stop codons. Absence of TDRD6 significantly impairs one specific mechanism of NMD, which depends on long 3’ untranslated regions of the transcripts. Thus, the CB component TDRD6 acts in the assembly of the NMD machinery in the CB.

## Introduction

During mammalian gametogenesis, substantial changes in chromosome structure and in gene expression profiles occur. Male germ cell transcription and transcriptomes have been studied quite extensively. Some transcriptome studies concern the whole testis, and thus represent combined somatic and germ cell data, which limits germ cell-specific conclusions. Other transcriptome reports describe meiosis- and/or postmeiosis-specific gene expression patterns (reviewed in [1]), some reports deal with the silencing of unsynapsed axial elements in early meiosis (reviewed in [2]), with the silencing associated with histone replacement by specific histone variants or by protamines (reviewed in [3]), with specific RNA species such as small RNA families (reviewed in [4]), with alternative splicing in germ cells (reviewed in [5]) or with transcription factors acting specifically in male germ cells (reviewed in [6]). Various RNA binding proteins were studied (reviewed in [7] and are mostly involved in processing of RNA species. Proper post-transcriptional processing of RNA molecules is essential for germ cell development and thus to producing gametes. For example, in late spermiogenesis, transcripts with short 3’ untranslated regions (UTRs) become preferred and are more stable [8] due to changes in 3’ UTR processing factors [9]. An example of such regulation is the Rnf4 transcript of which a long isoform is present in spermatocytes and a shorter isoform, due to truncation of the 3’ UTR at an upstream polyadenylation site, is present in spermatids [10]. The localization and regulation of mRNA translation is also specifically regulated. mRNAs coding for proteins important for the late stages of spermiogenesis, such as *Prm1* and *Prm2*, are synthesized in the early round spermatid stage, but reside in translationally repressed mRNP complexes. The transcripts become competent for active translation only during the later stages [11]. The apparent reason for such regulation is the transcriptional inactivity of the elongating spermatids due to the extensive nuclear condensation,i.e. transcripts are produced early and stored for later translation. RNA-rich structures termed “nuage’” or “granules” appear in spermatogenic cells and seem to orchestrate this peculiar post-transcriptional program.

The cytoplasmic presence of “germ granules” is a unique feature of germ cells is. Germ granules are RNA-rich, non-membranous cytoplasmic structures that have been proposed to play important roles in RNA post-transcriptional regulation [12]. On the basis of structural features and protein composition, the different germ granules of mammalian spermatogenic cells are designated, for example, chromatoid bodies (CBs) in spermatids and intermitochondrial cement (ICM) in spermatocytes [13]. The CBs appear first as fibrous and granulated material in the interstices of mitochondrial clusters and in the perinuclear area of pachytene spermatocytes. After meiosis, the CBs condense into one single lobulated, perinuclear granule in round spermatids and disassemble later during spermatid elongation. It has been shown that CBs contain mRNA, miRNA and piRNA species [14,15]. Proteins that participate in RNA transport such as KIF17b, miRISC (miRNA induced silencing complex) proteins such as the Argonaute family proteins AGO2, AGO3 and Dicer, proteins implicated in piRNA biogenesis and function such as MIWI and MILI [14,16], RNA helicases/binding proteins such as MVH(DDX4), UPF1, GRTH(DDX25) and PABP and RNA decaying factors such as SMG6 [17] were recently found in CBs. According to their molecular composition, CBs were proposed to function in translational repression, RNA silencing and mRNA storage. Thus, CBs may provide a platform for various RNA processing enzymes and processes, which are, however, little described.

The CBs are enriched also in TUDOR domain (TDRD) containing proteins. It was proposed that the TUDOR domains of these proteins provide interaction interfaces and create a scaffold to organize the CB structure [18]. The association of TDRD5, TDRD9 and TDRD7 with piRNA biogenesis enzymes such as MILI and MIWI is essential for piRNA biogenesis and retrotransposon mRNA silencing, and established a role of CBs in piRNA processing [19–21]. A role of CBs in splicing was suggested based on the observation that TDRD1 participates in complexes with snRNAs in the context of CBs [22]. Previous work by us [23] showed that TDRD6 is major component of CBs and is required for its architecture. Ablation of TDRD6 disrupts the CB structure and leads to developmental arrest at the round-to-elongated spermatid stage. Altered presence of miRNAs was observed in *Tdrd6*^*-/-*^ spermatocytes, but piRNA biogenesis and retrotransposon silencing were not affected.

However, whether TDRD6 is implicated in other mRNA metabolic processes that may occur within the CB was unknown. To gain insights into functions of TDRD6 and thus likely of the CB, we performed proteomics of purified CBs. Having identified UPF1 and UPF2 in the CB, which are key proteins in the nonsense mediated mRNA decay (NMD) pathway, we aimed at determining the contribution of TDRD6 to mRNA decay. Previous reports on processes associated with the 3’ end of mRNAs in spermatogenesis describe specific signals embedded in the 3’ UTR sequences or with individual proteins binding there (reviewed in [24,25]. However, the NMD pathway has not been described for mouse or human spermatocytes or spermatids. Processes and complexes that serve mRNA stability and function in germ cells are not sufficiently understood.

We show here that TDRD6 is essential for UPF1 localization to CBs and is critical for UPF1-UPF2 and UPF-MVH interactions. We report that a specific branch of NMD, the 3’ UTR length-triggered pathway, but not the downstream exon-exon junction dependent mode of NMD, is affected by absence of TDRD6 and thus CB distortion. We further show that association of some mRNAs with UPF1 is impaired in *Tdrd6*^*-/-*^ spermatids, perturbing mRNA processing. We suggest that in spermatids TDRD6 is required for the specific long 3’ UTR dependent NMD pathway, which most likely acts within the CB.

## Results

### TDRD6-dependent chromatoid body proteome

**Fig 1:**
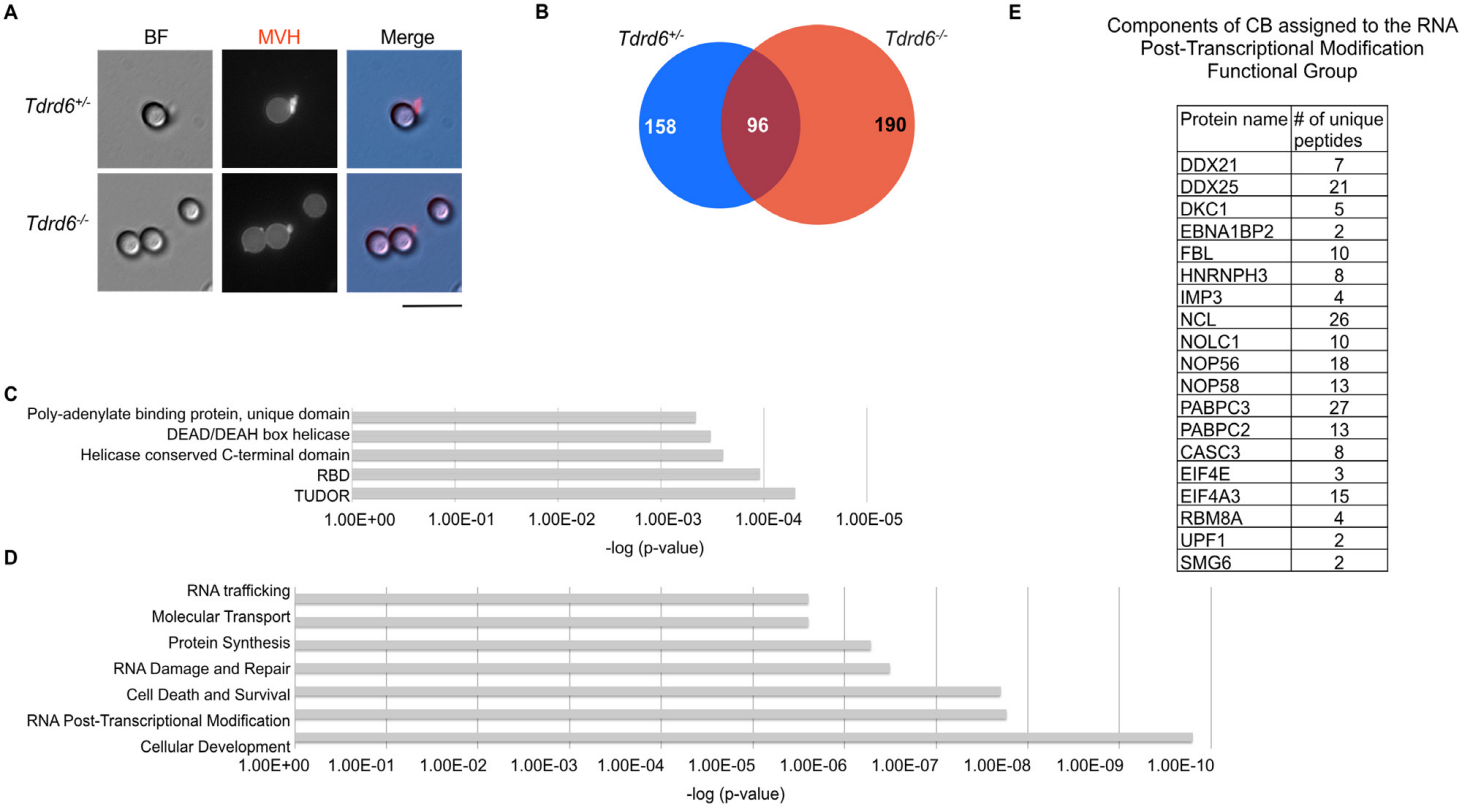
Proteomic analysis of CBs. **(A)** Immunofluorescence staining of *Tdrd6*^*+/-*^ and *Tdrd6*^*-/-*^ CBs attached on Dynabeads coupled with anti-MVH. CBs were visualized by anti-MVH staining. Signal obtained from Dynabeads is a result of anti-MVH coupling. Dynabeads can be visualized in the bright-field channel (BF). Scale bar 10 μm. **(B)** Venn diagram showing proteins identified in *Tdrd6*^*+/-*^ and *Tdrd6*^*-/-*^ CB preparations.**(C)** Bar plot showing the most enriched domains in 158 proteins found exclusively in *Tdrd6*^*+/-*^ CB preparation. The analysis was performed by DAVID 6.7 functional annotation tool and the results are ordered by p-value of enrichment. **(D)** Bar plot showing the most enriched functional groups according to Molecular and Cellular Function of 158 proteins found exclusively in *Tdrd6*^*+/-*^ CB preparation. The analysis was performed by IPA and the results are ordered by p-value of enrichment. **(E)** List of proteins of RNA Post-Transcriptional Modification functional group found enriched in *Tdrd6*^*+/-*^ CB preparation and the corresponding number of unique peptides identified by MS.

TDRD6 was proposed to play an architectural role in the assembly of CBs such as a scaffold protein. Morphological studies showed that in *Tdrd6* ^*-/-*^ spermatids, the CBs were found less compacted and of lower density [23]. We investigated the contribution of TDRD6 to CB composition by determining the protein constituents of CBs from *Tdrd6*^*+/-*^ and *Tdrd6* ^*-/-*^ spermatids. Based on a method described previously [15] we isolated CBs from adult *Tdrd6*^*+/-*^ and *Tdrd6* ^*-/-*^ testes. Testicular cell suspensions were chemically fixed to preserve the CB structures during the subsequent step of cell lysis in high stringency buffer. The lysates were centrifuged at low speed to acquire a CB-rich pellet. By immunostaining of MVH we could observe the presence of large, ring-like CB structures in *Tdrd6*^*+/-*^ samples, and in *Tdrd6* ^*-/-*^ samples smaller structures that may represent less compacted CBs as expected for in *Tdrd6* ^*-/-*^ cells or precursor building blocks of CBs (S1Aii Fig). Next, *Tdrd6*^*+/-*^ and *Tdrd6* ^*-/-*^ CBs were attached to anti-MVH Dynabeads and immunoprecipitated (Fig 1A). Similar efficiency of immunoprecipitation from *Tdrd6*^*+/-*^ and *Tdrd6* ^*-/-*^ samples was obtained as seen by immunoblotting of the preparations for MVH (S1B Fig). The immunoprecipitated *Tdrd6*^*+/-*^ and *Tdrd6* ^*-/-*^ CB samples were resolved in SDS-PAGE gels and subjected to mass spectrometric analysis. Substantial differences in protein content were observed in TDRD6-deficient CBs compared to controls. We found 286 proteins in *Tdrd6*^*-/-*^ CB preparations and 254 proteins in *Tdrd6*^*+/-*^ CBs (S3 Table). We reckon that TDRD6 is key to supporting a normal protein composition of CBs. Only 96 proteins are present in both samples and thus do not require TDRD6 for their assembly in CBs. Those 96 proteins were excluded form the further analysis and we focused on the 158 proteins enriched exclusively in *Tdrd6*^*+/-*^ CB samples (Fig 1B). The 190 proteins not present in unperturbed CBs but present in absence of TDRD6 may result from aberrant associations with MVH made possible by the removal of TDRD6.

We further analyzed the proteome data by focussing on proteins whose presence in the CB depends upon TDRD6. We performed Domain and GO term analysis through the DAVID platform [26] and QIAGEN’s Ingenuity Pathway Analysis (IPA, QIAGEN Redwood City). Analysis of enriched domains revealed that proteins with TUDOR domains, RNA binding domains, or helicase domains are enriched in the *Tdrd6*^*+/-*^ CBs (Fig 1C) confirming previous studies [15]. The most represented GO terms and thus molecular and cellular functions are Cell Death and Survival, Cell Development, Protein Synthesis and RNA metabolism such as RNA trafficking, RNA Damage and Repair, RNA Post-Transcriptional Modification (Fig 1D). Thus, the CB localization of proteins bearing RNA binding domains and of proteins that participate in RNA post-transcriptional modification mechanisms was confirmed and is prominently affected by the loss of TDRD6.

Among the proteins identified in the “RNA Post-Transcriptional Modification” group are DEAD box RNA helicases (DDX21, DDX25), snoRNP related proteins (IMP3, DKC1, NOP56, NOP58), rRNA metabolism related proteins (EBNA1BP2, FBL, NCL), pre mRNA binding protein (HNRNPH3), exon junction complex proteins (EIF4A3, CASC3, RBM8A) and RNA decaying enzymes (UPF1, SMG6) (Fig 1E). UPF1 is a key factor of nonsense mediated mRNA decay (NMD). Initially the NMD pathway was considered as a quality control system that recognizes and degrades aberrant mRNAs with truncated open reading frames (ORF) due to the presence of a premature termination codon (PTC) [27]. However, recent studies demonstrated a general role of NMD in post-transcriptional regulation of non-aberrant mRNAs. Upstream ORF (uORF), introns in 3’ UTR and long 3’ UTRs have been identified as features that activate NMD [28]. UPF1 was found to be enriched at long 3’ UTR sequences [29,30] and increased association of UPF1 with the 3’ UTR triggers the decay of the mRNA [31]. Along with UPF1, UPF2 supports the decay of mRNAs with long 3’ UTR [32]. UPF1 was previously shown to be a component of the CB [17] and the fact that we identified UPF1 in intact, but not in disturbed CBs, motivated us to further analyze UPF1 and its partner UPF2 in spermatids.

Generally, very little is known about the presence and function of UPF complexes in germ cell development. To address whether the expression of *Upf* genes is developmentally regulated in the testis, we isolated and analyzed RNA from testis of successive days post partum, i.e. during the first wave of spermatogenesis and spermiogenesis (S2A Fig). *Upf1* and *Upf2* are weakly expressed in neonatal testes and their expression increases during the development of spermatocytes and spermatids. *Upf1* and *Upf2* expression increases moderately in meiotic cells, which were identified by *Hormad1* expression at day 10 postpartum (pp) [33]. Later *Upf1* and *Upf2* expression peaks and coincides with high *Tdrd6* expression at day 22 pp, which marks the appearance of early round spermatids [23]. *Upf1* and *Upf2* expression remain at high levels as round spermatids differentiate to elongated spermatids marked by *Prm2 e*xpression form 26 pp onwards. These data suggest that UPF complexes may play an hitherto undescribed role in the late meiotic and postmeiotic stages of spermatogenesis.

The parallel expression of *Upf1*, *Upf2* and *Tdrd6* led us to test whether the levels of *Upfs* are affected by TDRD6 deficiency. We isolated mRNA (S2B Fig) and protein extracts (S2C Fig) from *Tdrd6*^*+/-*^ and *Tdrd6*^*-/-*^ spermatids and compared mRNA and protein expression of UPFs. The absence of TDRD6 and thus the disruption of CBs did not affect levels of UPFs protein or mRNA.

### TDRD6 associates with MVH, UPF1 and UPF2 and supports UPF1-UPF2 interaction

**Fig 2:**
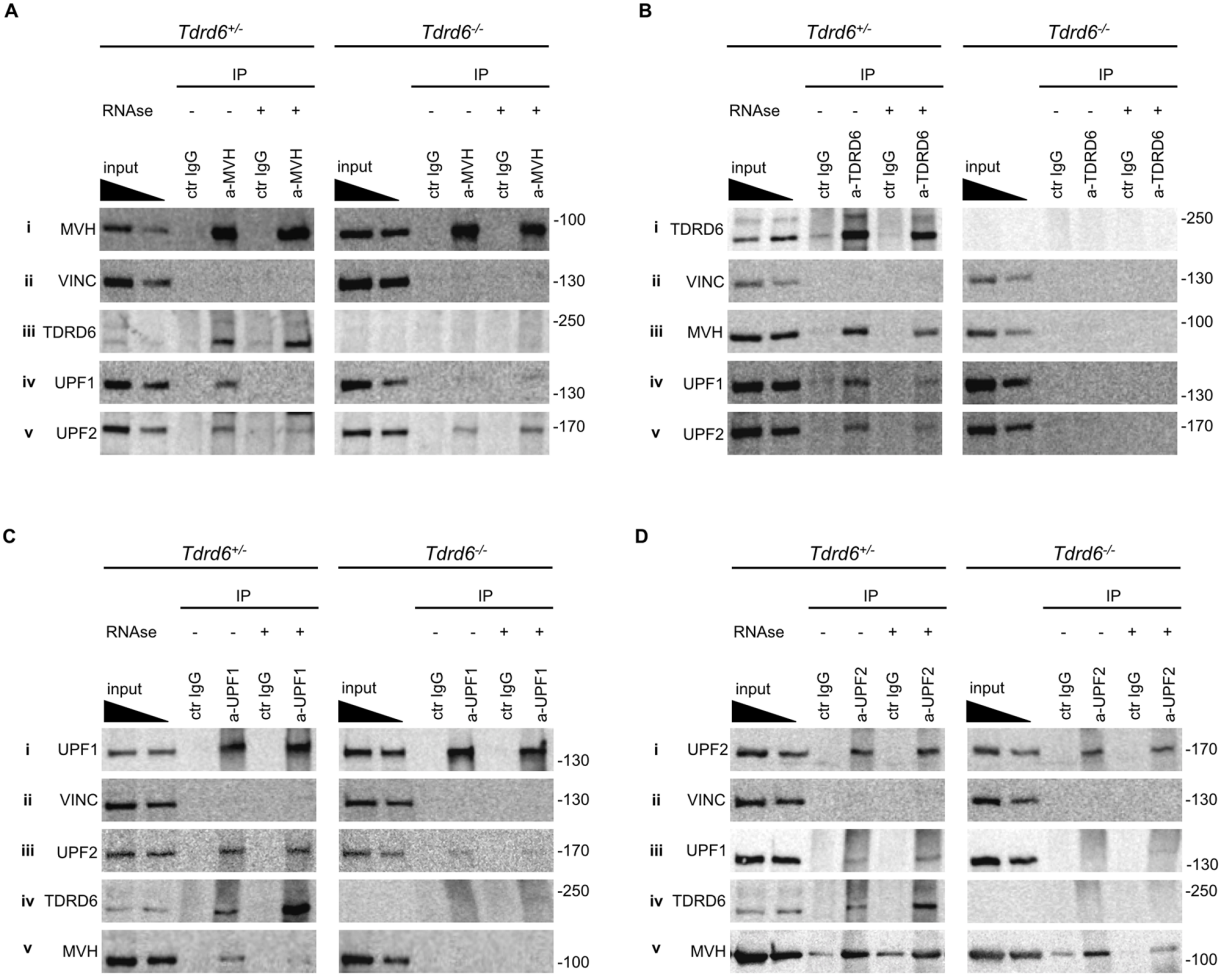
Associations of UPF proteins with CB components. **(A,B,C,D)** Cell lysate from *Tdrd6*^*+/-*^ (left) and *Tdrd6*^*-/-*^ (right) round spermatids was precipitated with antibodies specific for **(A)** MVH **(B)** TDRD6 **(C)** UPF1 and **(D)** UPF2. Irrelevant rabbit IgG was used for immuno-precipitation (IP) control, RNAse A treatment was included as indicated. IP proteins were separated by SDS-PAGE and transferred to nitrocellulose membranes. Membranes were probed with anti-TDRD6, anti-MVH, anti-UPF1, anti-UPF2. Probing with anti-VINC serves as negative co-IP control. Inputs represent 10% and 5% of the sample used for immuno-precipitation. Molecular weight in kilodalton is noted on the right side of each blot. All images are representative from at least 3 independent IP experiments.

We investigated the associations—direct or indirect—of TDRD6, MVH, UPF1 and UPF2 in *Tdrd6*^*+/-*^ and *Tdrd6*^*-/-*^ by co-immunoprecipitation in the presence or absence of RNAse A treatment. RNAse A treatment efficiency was assessed by RNA electrophoresis of the flow-through sample of the IPs (S2D Fig). We investigated the interaction between MVH, UPF1 and UPF2 by performing MVH immuno-precipitation (IP) (Fig 2Ai). Vinculin (VINC) a membrane-cytoskeletal protein was used as a loading and negative co-IP control (Fig 2Aii). The previously reported *in vitro* interaction between TDRD6 and MVH [23] was recapitulated by co-IP from spermatids and was independent of RNAseA inclusion (Fig 2Aiii). Reverse IP of TDRD6 (Fig 2Bi) showed also that TDRD6 associated with MVH (Fig 2Biii) irrespectively of RNAse A treatment. UPF1 co-IP with MVH (Fig 2Aiv) was observed specifically only in *Tdrd6*^*+/-*^ spermatids but not in the *Tdrd6*^*-/-*^ spermatids or in IgG control IPs and was dependent on RNA. Reverse IP of UPF1 (Fig 2Ci) showed also its association with MVH (Fig 2Cv), but only when TDRD6 and intact RNA were present. UPF2 co-immunoprecipitated with MVH (Fig 2Av) irrespectively of the genotype and RNAse A treatment and the reverse IP of UPF2 (Fig 2Di) demonstrated its association with MVH (Fig 2Dv). Thus, only UPF1, but not UPF2, requires a TDRD6-supported, intact CB for its association with the key CB component MVH.

Since we observed differential association of UPF1 and MVH in *Tdrd6*^*+/-*^ and *Tdrd6*^*-/-*^ spermatids, we investigated the interaction between TDRD6, UPF1 and UPF2 by performing TDRD6 IP (Fig 2Bi). UPF1 co-immunoprecipitated with TDRD6 (Fig 2Biv) specifically only in *Tdrd6*^*+/-*^ spermatids but not in the *Tdrd6*^*-/-*^ spermatids or in IgG control IPs. Reverse IP of UPF1 (Fig 2Ci) showed also its association with TDRD6 (Fig 2Civ). UPF2 co-immunoprecipitated with TDRD6 (Fig 2Bv) and the reverse IP of UPF2 (Fig 2Di) demonstrated its association with TDRD6 (Fig 2Div). The interactions of TDRD6 and UPF1 or UPF2 are resistant to RNAse A treatment. This data suggested the involvement of TDRD6 in complexes containing MVH, UPF1 and UPF2.

UPF1 binds directly to UPF2 via an UPF2-interacting domain [34], but upon IP of UPF1 from *Tdrd6*^*+/-*^ and *Tdrd6*^*-/-*^ round spermatids (Fig 2Ci), UPF2 was found to co-immunoprecipitate only in the *Tdrd6*^*+/-*^ samples, UPF1 interaction with UFP2 is almost entirely abrogated upon loss of TDRD6 (Fig 2Ciii). Confirming these results, IP of UPF2 from *Tdrd6*^*+/-*^ and *Tdrd6*^*-/-*^ round spermatids (Fig 2Di) showed that UPF2 associated with UPF1 in the *Tdrd6*^*+/-*^ sample but hardly in absence of TDRD6 (Fig 2Diii). UPF1-UPF2 association in *Tdrd6*^*+/-*^ samples was not affected by the presence of RNAse A as expected. In conclusion, the absence of TDRD6, accompanied by distortion of CB structure, prevented UPF1-MVH and UPF1-UPF2 interactions.

### Localization of UPF1 and UPF2 in the CB of round spermatids

Given the distinctly TDRD6-dependent associations of UPF1 and UPF2 shown above, the localization of UPFs in germ cells was determined by staining meiotic and postmeiotic cells with antibodies against UPF1 and UPF2. The localization of UPF proteins has been extensively investigated in mammalian cell lines where UPF1 is mainly cytoplasmic [35], but a fraction of UPF1 resides in the nucleus where it promotes DNA replication, S phase progression [36] and telomere stability [37]. More recently it was shown that UPF proteins localize to P-bodies in mammalian cells [38]. UPF2 is a cytoplasmic protein [35].

**Fig 3:**
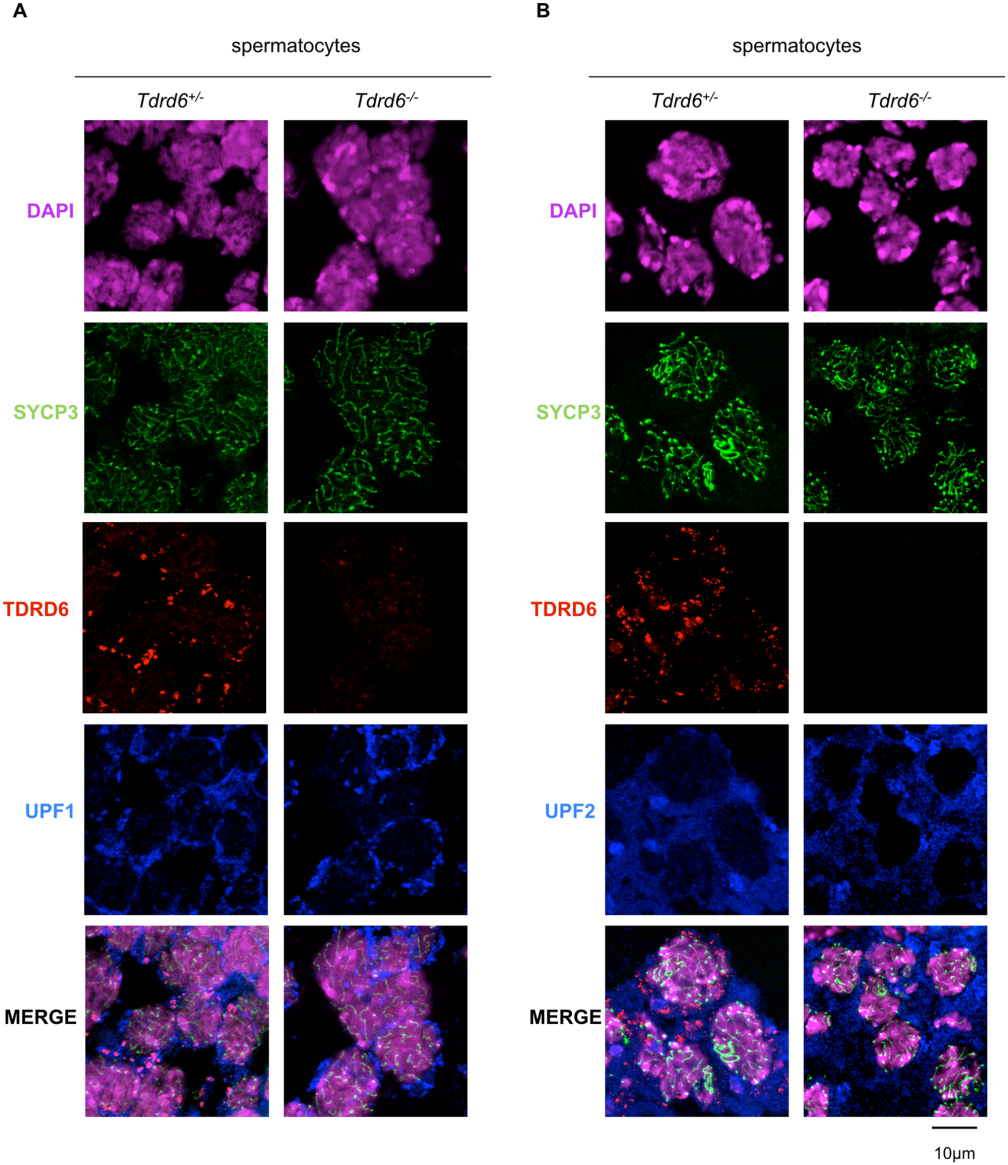
Localization of UPF proteins in primary spermatocytes. Immunofluorescence staining of *Tdrd6*^*+/-*^ and *Tdrd6*^*-/-*^ testes. Frozen sections of 18 dpp testes were stained with anti-SYCP3 (green), anti-TDRD6 (red), anti-UPF1 (blue) in **(A)** anti-UPF2 (blue) in **(B)**. DAPI (magenta) marks the nuclei. Scale bar 10 μm. Images are representative from 3 independent immunofluorescence experiments.

In meiosis I spermatocytes, positive for SYCP3, UPF1 localized to the perinuclear space of the cytoplasm and there was hardly any staining observed in the nucleus (Fig 3A). UPF2 was distributed in some clusters throughout the cytoplasm (Fig 3B). No apparent co-localization with TDRD6 was detected suggesting no participation in the precursor structures of CB in meiotic cells. The localization pattern of UPFs in meiotic cells remained unaffected by the loss of TDRD6 (Fig 3A and 3B).

**Fig 4:**
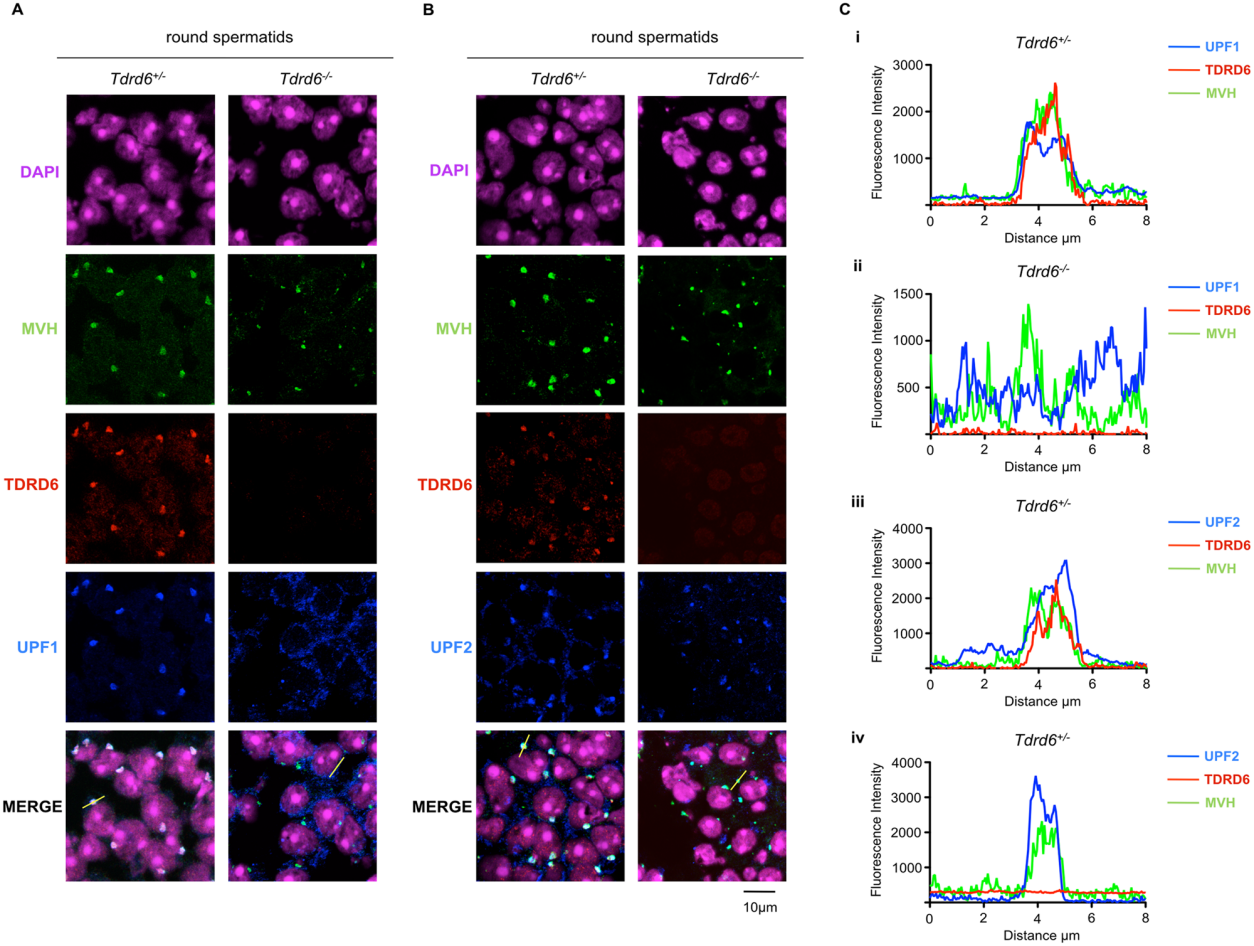
Localization of UPF proteins in round spermatids. Immunofluorescence staining of *Tdrd6*^*+/-*^ and *Tdrd6*^*-/-*^ testes. Frozen sections of 26 dpp testes were stained with anti-MVH (green), anti-TDRD6 (red), anti-UPF1 (blue) in **(A)** anti-UPF2 (blue) in **(B)**. DAPI (magenta) marks the nuclei. Scale bar 10 μm. Images are representative from 3 independent immunofluorescence experiments. **(C)** Line graphs showing the immunofluorescence intensity profile along the freely positioned line of MVH (green), TDRD6 (red) and UPF1 (blue) in *Tdrd6*^*+/-*^ **(i)**; MVH (green), TDRD6 (red) and UPF1 (blue) in *Tdrd6*^*-/-*^ **(ii)**; MVH (green), TDRD6 (red) and UPF2 (blue) in *Tdrd6*^*+/-*^ **(iii)**; MVH (green), TDRD6 (red) and UPF2 (blue) in *Tdrd6*^*-/-*^ **(iv).**

In *Tdrd6*^*+/-*^ mice, UPF1 was absent form the cytoplasm of round spermatids and was exclusively concentrated in CBs where it co-localized with MVH and TDRD6 (Fig 4A and 4Ci). However, in *Tdrd6*^*-/-*^ round spermatids UPF1 failed to co-localize with MVH positive foci, i.e. with the distorted CBs found in *Tdrd6*^*-/-*^ round spermatids, and remained diffuse in the perinuclear cytoplasm (Fig 4A and 4Cii). This suggested that TDRD6-positive, undistorted CBs are required for UPF1 re-localization from the cytoplasm of meiotic cells to the CBs of round spermatids. On the other hand, UPF2 (Fig 4B, 4Ciii and 4Civ) primarily co-localized with MVH in *Tdrd6*^*+/-*^ and *Tdrd6*^*-/-*^ CBs. UPF2 is a newly identified component of CBs. 100% (n = 67) and 97% (n = 76) of *Tdrd6*^*+/-*^ CBs scored contain UPF1 and UPF2, respectively. In *Tdrd6*^*-/-*^ round spermatids 0% (n = 41) of CBs contained mUPF1, while mUPF2 localized to 86% (n = 72) of *Tdrd6*^*-/-*^ CBs (S3 Fig). This indicated a TDRD6 independent manner of localization of UPF2 to CBs, although CB presence of these proteins was slightly affected probably by the distorted architecture of the *Tdrd6*^*-/-*^ CBs.

### Downstream exon-exon junction (dEJ) stimulated NMD does not require TDRD6 or intact CB

UPF1 is a key factor of nonsense mediated mRNA decay (NMD). Initially the NMD pathway was considered as a quality control system that recognizes and degrades aberrant mRNAs with truncated open reading frames (ORF) due to the presence of a premature termination codon (PTC) [27]. PTCs can arise from aberrant splicing events, 5’ UTR upstream open reading frames (uORFs) or by mutations. In principle, a termination codon residing more than ~55 nucleotides upstream of an exon-exon junction complex is considered a PTC and the transcript is a likely target for the so called downstream exon-exon junction stimulated (dEJ) NMD [27]. We hypothesized that mis-localization of UPF1 and failing interaction of UPF1 with UPF2 in the *Tdrd6*^*-/-*^ strain would lead to accumulation of NMD sensitive transcripts in *Tdrd6*^*-/-*^ round spermatids. To test whether the dEJ mode of NMD was affected by loss of TDRD6, we generated the mRNA profiles of germ cell populations enriched for round spermatids of *Tdrd6*^*+/-*^ and *Tdrd6*^*-/-*^ mice by deep sequencing. The MACS-purified population was more than 95% positive for expression of the marker hCD4 in both genotypes (S4A Fig), and the hCD4 is expressed at the same levels in *Tdrd6*^*+/-*^ and *Tdrd6*^*-/-*^ cells (S4B Fig). The preparations enriched for round spermatids contained approximately 70% round spermatids in both *Tdrd6*^*+/-*^ and *Tdrd6*^*-/-*^ samples (S4C Fig), and the fraction of primary and secondary spermatocytes was about the same. We used 4 biological replicates, i.e. round spermatid samples of four individual animals per genotype and acquired over 250 million RNA seq reads (S5 Fig). We aligned the RNA sequence reads with TopHat, assembled transcripts with Cufflinks and annotated them using Ensemble v67 [39–41]. Expression analysis was performed with Cuffdiff with a FDR of 0.1. We used 2 different approaches to classify transcripts with PTCs, which are putative dEJ NMD targets to be further analyzed. In the first approach, the mouse annotation of Ensembl v67 was used for the classification of the transcripts. Transcripts which had the biotype “Nonsense Mediated Decay” were extracted from the complete data set and used for the subsequent analysis. Here, if the coding sequence of a transcript finishes >50 bp from a downstream splice site, it is tagged as a putative NMD sensitive transcript. In the second approach, SpliceR version 1.12.0 was used for the annotation of transcripts with PTC [42]. We used the Cufflinks results files for SpliceR and filtered the isoforms with the setting "expressedIso" and "isoOK" within SpliceR. Furthermore SpliceR requires CDS information, which was retrieved with SpliceR internal function from UCSC. Annotation of transcripts was done with “annotatePTC” and transcripts were extracted, which were set to PTC equals TRUE. These transcripts were used for the comparison. There are 564 dEJ NMD sensitive transcripts identified only by Ensemble v67 database, 2004 transcripts identified only by the SpliceR pipeline and 1268 identified by both ways (S6A Fig).

**Fig 5:**
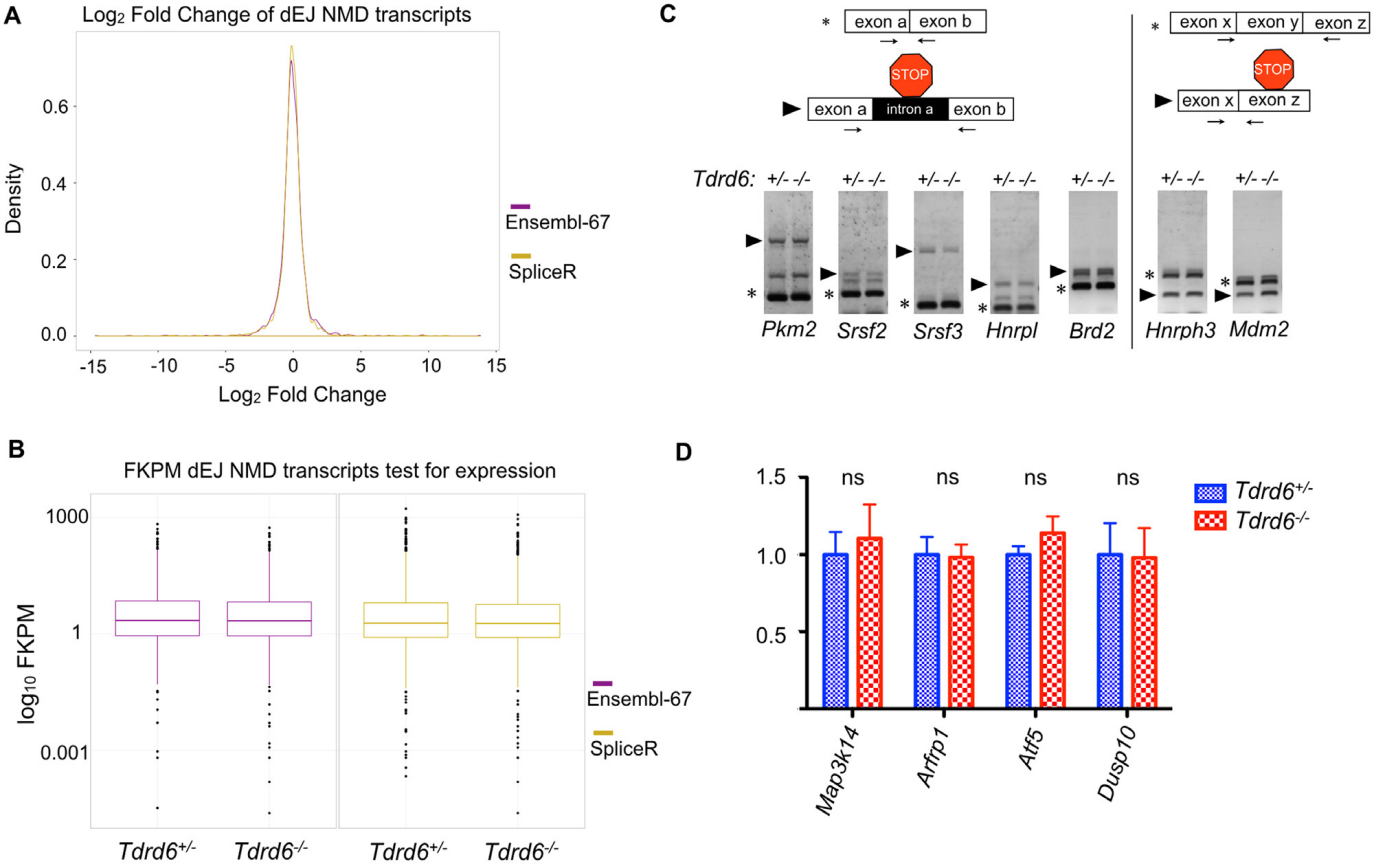
Analysis of dEJ NMD pathway. **(A)** Line Graph showing the distribution of expression changes for transcripts marked as putative dEJ NMD targets by Ensemble v67 (magenta line) and SpliceR (yellow line) between the two conditions (*Tdrd6*^*+/-*^ and *Tdrd6*^*-/-*^) in fold change (x axis; log_2_ values). **(B)** Box plot showing the expression values for transcripts marked as putative dEJ NMD targets by Ensemble v67 (magenta) and SpliceR (yellow) (y axis; log_10_ scale) for *Tdrd6*^*+/-*^ and *Tdrd6*^*-/-*^ given in fragments per kilobase per million sequenced reads (FPKM). **(C)** Top left: Schematic representation of a normal splicing event marked by asterisk and PTC upon intron inclusion event marked by arrowhead. Arrows represent primer pairs used in the assay below. Bottom left: RT-PCR analysis of *Pkm2*, *Srsf2*, *Srsf3*, *Hnrpl* and *Brd2* transcripts with known aberrant intron inclusion event. Top right: Schematic representation of a normal splicing event marked by asterisk and PTC upon exon exclusion event marked by arrowhead. Arrows represent primer pairs used in the assay below. Bottom right: RT-PCR analysis of *Hnrph3* and *Mdm2* transcripts with known aberrant exon exclusion event. Pictures are representative from 2 independent experiments. **(D)** RT-qPCR analysis of *Map3k14*, *Arfrp1*, *Atf5*, and *Dusp10* (known NMD sensitive mRNAs due to 5’ uORFs presence) mRNA expression in *Tdrd6*^*+/-*^ (blue bars) and *Tdrd6*^*-/-*^ (red bars) round spermatids. Results are presented in terms of a fold change after normalizing mRNA levels with *β-actin* mRNA level. Each value represents the mean of three independent experiments. ns not significant p value>0.1.

1,520 (82%) out of 1,832 dEJ NMD sensitive transcripts by Ensemble v67 have a log2 fold change between -1 and 1 and 1,089 (59%) transcripts have a log2 fold change between -0.5 and 0.5 (Fig 5A). Similarly 2780 (85%) out of 3272 dEJ NMD sensitive transcripts by SpliceR analysis have a log2 fold change between -1 and 1 and 2031 (62%) transcripts have a log2 fold change between -0.5 and 0.5 (Fig 5A) indicating normal regulation of dEJ NMD transcripts between *Tdrd6*^*+/-*^ *and Tdrd6*^*-/-*^ round spermatids. Next, we looked at the expression values measured in FPKM for the dEJ NMD sensitive transcripts in both genotypes (Fig 5B). A Wilcoxon-Mann-Whitney test (p-value = 0.4789 for the dEJ NMD sensitive transcripts by Ensemble v67 and p-value = 0.8998 for the dEJ NMD sensitive transcripts by SpliceR analysis) showed that there is no difference in the FPKM values of the dEJ NMD sensitive transcripts between the TDRD6-proficient and -deficient samples.

We further examined a number of known NMD substrates to validate the high throughput analysis. Abnormal splicing events such as intron inclusion, exon skipping and splicing downstream of a normal termination codon can induce the dEJ mode of NMD. During an intron inclusion event, a PTC can be introduced either because it resides in the included intron or is generated due to frameshift of the physiological ORF. In an exon skipping event a frameshift of the ORF might produce a PTC. We tested PTC generation by intron inclusion and exon skipping events that characterized for specific transcripts in other murine tissues [43]. Performing RT-PCR using specific primers (arrows), which span intron inclusion events for *Pkm2*, *Srsf2*, *Srsf3*, *Hnrpl*, *Brd2* and exon skipping events for *Hnrnph3* and *Mdm2*, we investigated NMD sensitive transcript variants (marked by arrowheads). NMD sensitive or NMD resistant variants showed the same levels in *Tdrd6*^*+/-*^ and *Tdrd6*^*-/-*^ samples (Fig 5C).

*Auf1* mRNA can also be used as a marker of NMD efficiency due to its unusual 3’ UTR architecture [44]. Splicing of exon 9 and exon 10 generates an exon junction more than 50 nt downstream of the normal termination codon, producing NMD sensitive transcript variants II and III (S6B Fig and [44]). We designed specific primers to map different splicing events and found that splicing events producing the NMD sensitive transcripts II and III occur largely the same in *Tdrd6*^*+/-*^ and *Tdrd6*^*-/-*^ samples (S6C Fig).

Finally, uORFs of a transcript would lead to premature translational termination and subsequent NMD. We compared the expression of transcripts with uORFs such as *Atf5*, *Map3k14*, *Arfp1* and *Dusp10*, which were previously shown to be recognized by NMD in other cell types [45,46] by RT-qPCR. We found no difference in their expression levels in *Tdrd6*^*+/-*^ and *Tdrd6*^*-/-*^ round spermatids (Fig 5D). Together these data showed normal function of the downstream exon-exon junction dependent mode of NMD in TDRD6 deficient spermatids.

### mRNAs with long 3’ UTR accumulate in TDRD6 deficient, CB-disrupted round spermatids

**Fig 6:**
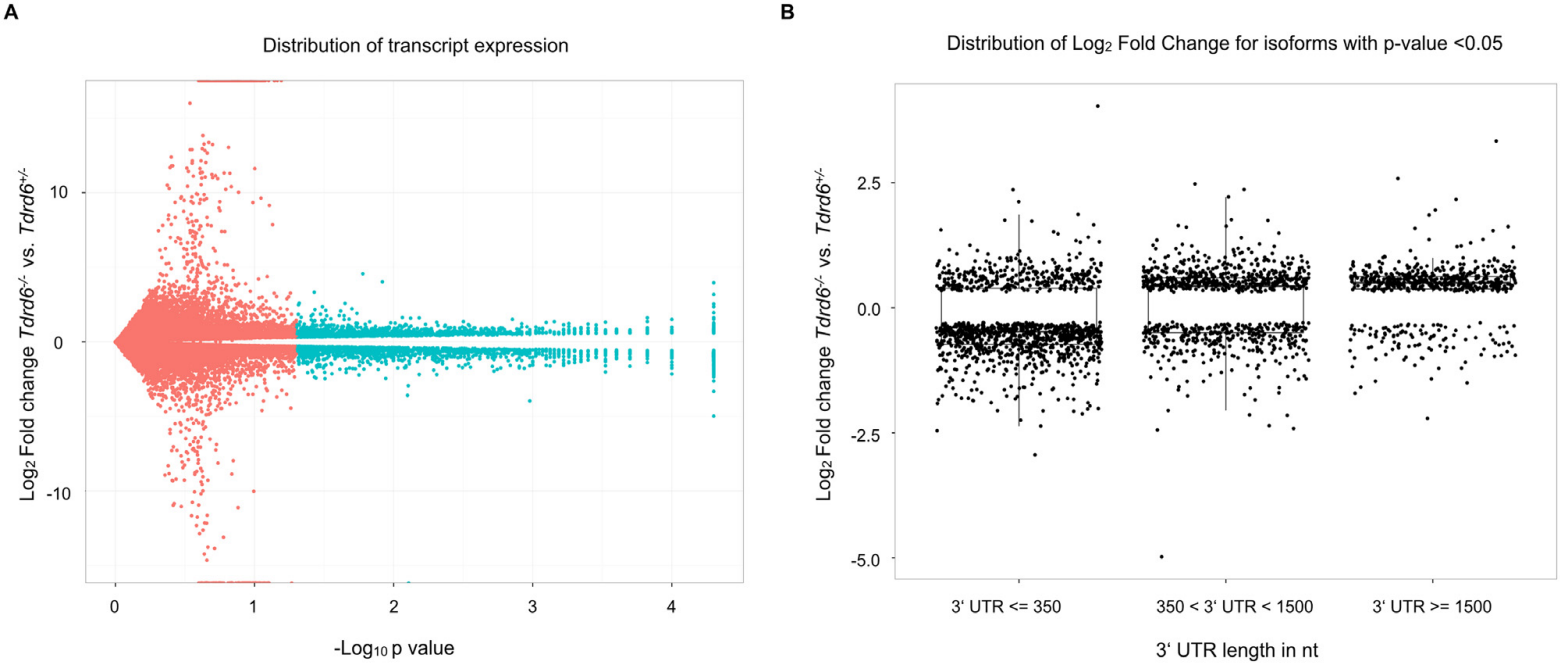
Transcriptomic alterations in CB deficient round spermatids. **(A)** Volcano plot showing the global transcriptional changes in *Tdrd6*^*-/-*^ vs. *Tdrd6*^*+/-*^ round spermatids. Each dot represents one transcript. The log_2_ fold change in *Tdrd6*^*-/-*^ versus *Tdrd6*^*+/-*^ is represented on the y-axis. Transcripts that differ significantly (p-value < 0.05; shown as negative log10 on the x-axis) between the conditions *Tdrd6*^*-/-*^ and *Tdrd6*^*+/-*^ are represented with blue dots. **(B)** Dot plot showing the distribution of fold change of transcripts in *Tdrd6*^*-/-*^ versus *Tdrd6*^*+/-*^ (y axis; log_2_ values) for the significantly differentially expressed transcripts by 3’ UTR length (x axis; length given in nucleotides).

Although NMD was initially characterized in PTC dependent mRNA degradation as a quality control mechanism, there is evidence that NMD is implicated in the metabolism of normal mRNAs. A well studied feature of mRNAs which can elicit NMD is the long 3‘ UTR. UPF1 was found to be enriched at long 3’ UTR sequences [29,30] and increased association of UPF1 with the 3’ UTR triggers the decay of the mRNA [31] in an UPF2 and SMG6 dependent way [32]. Since we found TDRD6 to associate with UPF1 and UPF2, we assessed the effect of TDRD6 deficiency on the general mRNA transcriptome. We analyzed the expression of normal mRNAs in the transcriptome data described above, derived from germ cell populations enriched for round spermatids from *Tdrd6*^*+/-*^ and *Tdrd6*^*-/-*^ mice. We aligned the RNA seq reads with TopHat, assembled transcripts with Cufflinks and annotated them using Ensemble v67 [39–41]. Expression analysis was performed with Cuffdiff with a FDR of 0.1 and we found 2704 transcripts to be significantly (p-value <0.05) mis-regulated in absence TDRD6 and thus of intact CBs. More specifically, 1375 were up-regulated and 1329 down-regulated in *Tdrd6*^*-/-*^ round spermatids (Fig 6A and S1 Table). Thus, TDRD6 is required for the presence of a proper mRNA repertoire in spermatids.

To further characterize the changes of the mRNA content of CB-disrupted spermatids, we grouped mis-regulated transcripts with p-values <0.05 into 3 groups according to the length of their 3’ untranslated region (UTR): short 3’ UTR <350 nt, medium 3’ UTR >350 nt and <1500 nt and long 3’ UTR >1500 nt. We analyzed the log_2_ fold distribution of these groups of transcripts. We observed that the majority of mis-regulated transcripts (514, 82% out of 628) with a long 3' UTR had a positive log_2_ fold change, i.e. they are present at higher levels in the *Tdrd6*^*-/-*^ compared to the *Tdrd6*^*+/-*^ round spermatids. The distribution of positive and negative log_2_ fold change of mis-regulated transcripts was not significantly altered for short and medium 3‘UTR length groups, but the log_2_ fold distribution of transcripts with long 3’ UTR, showing enrichement of upregulated transcripts in *Tdrd6*^*-/-*^ samples was statistically different from the others (Wilcoxon-Mann-Whitney test p-value <2.2^−16^) (Fig 6B and S2 Table). We conclude that the mis-regulation of mRNAs in TDRD6 deficient, CB-disrupted spermatids correlates with an accumulation of transcripts carrying long 3’ UTRs.

Significantly mis-regulated transcripts with long 3’ UTRs >1500 nt correspond to 628 genes. 288 (46%) genes have a single transcript with long 3 UTR and 340 (54%) genes have multiple transcripts and transcripts with long 3’ UTRs among them (S6D Fig). These 340 genes code for 1176 putative transcript isoforms. These putative isoforms include the 340 long 3’ UTR transcripts tested previously, but in addition there are 167 isoforms that have 3’ UTRs shorter than 1500 nt and for the rest there is no reliable information on the 3’ UTR length. From the 167 short 3 ‘UTR isoforms of the genes with mis-regulated long 3’ UTR transcript isoforms, there are 15 transcripts, corresponding to 14 genes, showing a significant mis-regulation, while the large majority of 152 transcripts remained unchanged. Thus loss of TDRD6 affects specifically the long 3’ UTR isoforms of genes with multiple isoforms with different 3’ UTR lengths.

Further, we used the database of EMBL-EBI Expression Atlas and looked the expression analysis of different murine tissues. There are 10760 genes expressed in testis above a standard expression cutoff value of 0.5. We consider a transcript to be testis-specific when its expression is 10 times higher in testis than in any other tissue examined. There are 1851 genes that fall in this group. Of the 628 mis-regulated transcripts with long 3’ UTR there are 35 which can be considered testis specific (6%). 514 transcripts with long 3‘ UTRs are higher in the *Tdrd6*^*-/-*^ round spermatids and 15 of them are testis-specific (3%). 113 transcripts with long 3’ UTRs are lower in the *Tdrd6*^*-/-*^ round spermatids and 20 of them are testis-specific (17%).

### TDRD6 deficiency impairs long 3’ UTR-stimulated NMD by interfering with UPF1-mRNA binding

**Fig 7:**
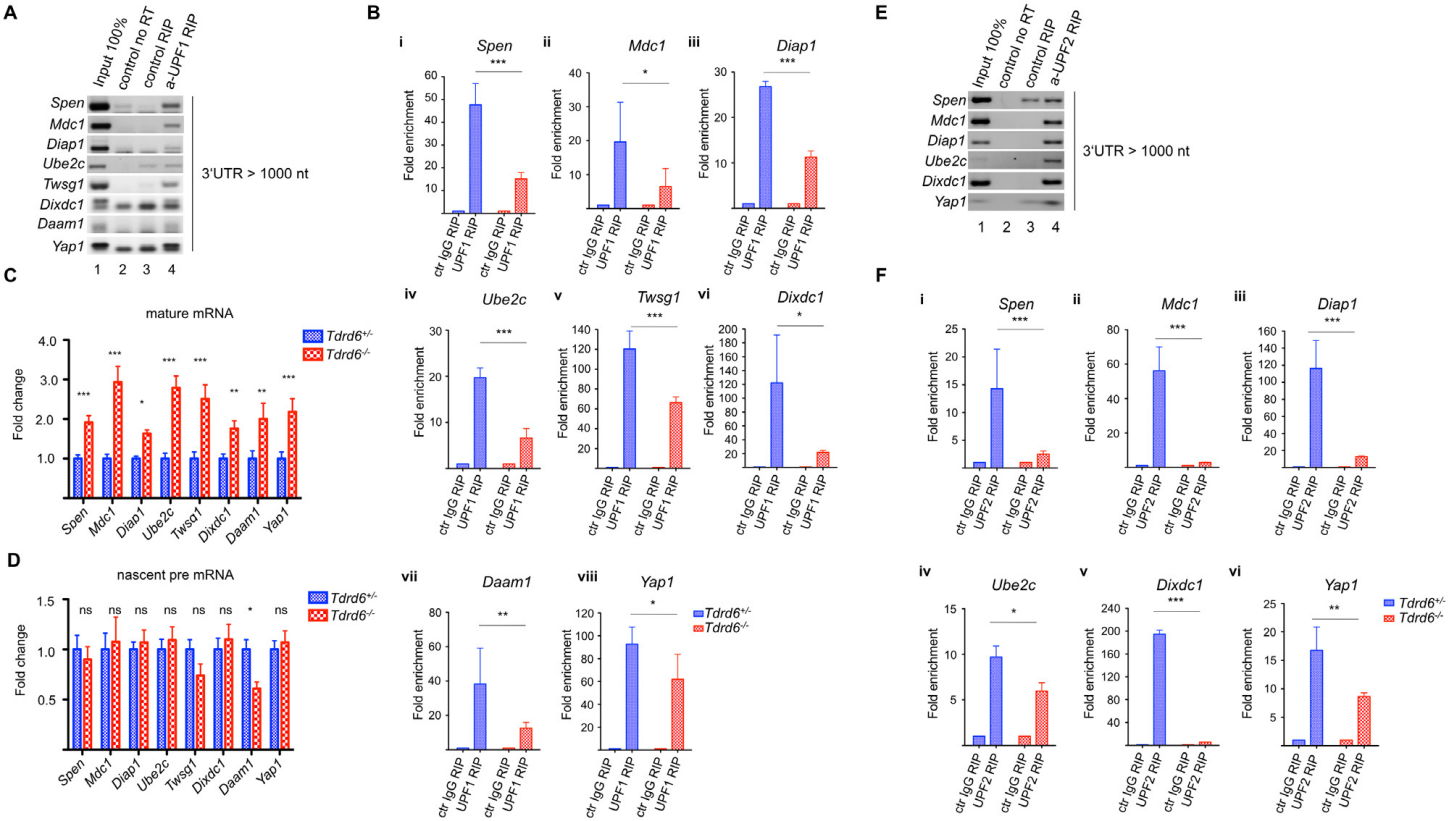
UPF1 and UPF2 association with mRNAs. **(A)** RT-PCR analysis of long 3‘UTR mRNAs i.e. *Spen*, *Diap1*, *Mdc1*, *Ube2c*, *Twsg1*, *Dixdc1*, *Daam1* and *Yap1* in UPF1-immunoprecipitated RNA from *Tdrd6*^*+/-*^ round spermatids. RNAs were immunoprecipitated from round spermatids lysate either with irrelevant goat IgG (lane 3) of anti-UPF1 (lane 4). RNA was isolated from the total lysate (lane 1) and reverse transcribed except from lane 2. **(B)** RT-qPCR analysis of *Spen* (i), *Mdc1* (ii), *Diap1* (iii), *Ube2c* (iv), *Twsg1* (v) *Dixdc1* (vi), *Daam1* (vii) and *Yap1* (viii) in UPF1-immunoprecipitated RNA from *Tdrd6*^*+/-*^ (blue bars) and *Tdrd6*^*-/-*^ (red bars) round spermatids. Bars represent the fold enrichment (mean and standard deviation (SD) n = 3) of different mRNA species isolated by anti-UPF1 RIP over the control RIP from *Tdrd6*^*+/-*^ and *Tdrd6*^*-/-*^ round spermatids after normalization to the respective input. **(C)** RT-qPCR analysis *Spen*, *Mdc1*, *Diap1*, *Ube2c*, *Twsg1*, *Dixdc1*, *Daam1* and *Yap1* mature mRNA expression in *Tdrd6*^*+/-*^ (blue bars) and *Tdrd6*^*-/-*^ (red bars) round spermatids. Results are presented in terms of a fold change after normalizing mRNA levels with *β-actin* mRNA level. Each value represents the mean of three independent experiments. **(D)** RT-qPCR analysis *Spen*, *Mdc1*, *Diap1*, *Ube2c*, *Twsg1*, *Dixdc1*, *Daam1* and *Yap1* pre mRNA expression in *Tdrd6*^*+/-*^ (blue bars) and *Tdrd6*^*-/-*^ (red bars) round spermatids. Results are presented in terms of a fold change after normalizing mRNA levels with *β-actin* mRNA level. Each value represents the mean of three independent experiments. **(E)** RT-PCR analysis of long 3‘UTR mRNAs i.e. *Spen*, *Diap1*, *Mdc1*, *Ube2c*, *Dixdc1* and *Yap1* in UPF2-immunoprecipitated RNA from *Tdrd6*^*+/-*^ round spermatids. RNAs were immunoprecipitated from round spermatids lysate either with irrelevant goat IgG (lane 3) of anti-UPF1 (lane 4). RNA was isolated from the total lysate (lane 1) and reverse transcribed except from lane 2. **(F)** RT-qPCR analysis of *Spen* (i), *Mdc1* (ii), *Diap1* (iii), *Ube2c* (iv), *Dixdc1* (v) and *Yap1* (vi) in UPF2-immunoprecipitated RNA from *Tdrd6*^*+/-*^ (blue bars) and *Tdrd6*^*-/-*^ (red bars) round spermatids. Bars represent the fold enrichment (mean and standard deviation (SD) n = 2) of different mRNA species isolated by anti-UPF1 RIP over the control RIP from *Tdrd6*^*+/-*^ and *Tdrd6*^*-/-*^ round spermatids after normalization to the respective input. * significant at p value<0.1, ** significant at p value<0.05, *** significant at p value<0.01, ns not significant p value>0.1.

UPF1 is an RNA helicase that can bind to all transcripts, although it preferentially associates with transcripts carrying long 3’ UTRs [30,47]. Our initial observation that mRNAs with long 3’ UTR tend to be up-regulated in CB distorted round spermatids led us to investigate particular mRNAs with 3’ UTR length >1000 nt with respect to UPF1 binding, mRNA levels and translational potential. We assessed the *in vivo* binding of UPF1 to selected mRNAs by performing anti-UPF1 RNA immunoprecipitation (RIP) from *Tdrd6*^*+/-*^ round spermatids, followed by RT PCR. We examined 9 transcripts that carried long 3’ UTRs >1000 nucleotides and 2 transcripts with 3’ UTRs <350 nucleotides as a negative control. Positive signals in the anti-UPF1 RIP RT-qPCR were obtained for transcripts with long 3’ UTR as *Spen* (3’ UTR length = 1016 nt), *Diap1* (3’ UTR length = 2244 nt), *Mdc1* (3’ UTR length = 2040 nt), *Ube2c* (3’ UTR length = 1598 nt), *Twsg1* (3’ UTR length = 3218 nt), *Dixdc1* (3’ UTR length = 3438 nt), *Daam1* (3’ UTR length = 2478 nt), *Yap1* (3’ UTR length = 2497 nt) (Fig 7A) and *Wnt3* (3’ UTR length = 1889 nt) (S7A Fig) and for transcripts with short 3’ UTR as *Ecsit* (3’ UTR length = 78 nt), *Prss51* (3’ UTR length = 119 nt) (S7A Fig), suggesting that UPF1 binds *in vivo* to all these transcripts.

Next, we expanded the analysis of mRNA to UPF1 binding in the *Tdrd6*^*+/-*^ versus *Tdrd6*^*-/-*^ round spermatids. Significantly decreased binding to UPF1 in *Tdrd6*^*-/-*^ round spermatids was observed for transcripts with long 3’ UTR such as *Spen*, *Mdc1*, *Diap1*, *Ube2c*, *Twsg1*, *Dixdc1*, *Daam1* and *Yap1* (Fig 7Bi–7Bviii). To assess the effect of impaired UPF1-mRNA binding on the mRNA levels, we performed RT-qPCR. The presence of mature mRNAs of *Spen*, *Mdc1*, *Diap1*, *Ube2c*, *Twsg1*, *Dixdc1*, *Daam1* and *Yap1*, *i*.*e*. the transcripts with long 3’ UTR showing decreased association with UPF1 in *Tdrd6*^*-/-*^ samples, was increased 2- to 3-fold in *Tdrd6*^*-/-*^ round spermatids (Fig 7C). The pre-mRNA levels of these genes remained unchanged (Fig 7D), showing that the higher levels were not caused by increased transcription, but by increased stability. These results suggested that UPF1 binding to mRNAs carrying long 3’ UTR is perturbed upon TDRD6 deletion—and thus distortion of the CB and mis-localization of UPF1 –and correlates with increased mRNA stability possibly through decreased degradation.

We identified short 3’ UTR such as *Ecsit* and *Prss51* (S7Bi and S7Bii Fig) and long 3‘UTR such *Wnt3* (S7Bii Fig) to associate with UPF1 equally between *Tdrd6*^*+/-*^ and *Tdrd6*^*-/-*^ round spermatids. The expression levels of transcripts that show unaffected association with UPF1 among the different genotypes were mis-regulated both on the mature mRNA (S7C Fig) and nascent pre mRNA levels (S7D Fig) possibly through indirect mechanisms.

In RIP experiments using anti UPF2 antibody, we found that most of the mRNAs with long 3’ UTR that associate with UPF1 also associate with UPF2 in round spermatids (Fig 7E). The analysis of binding of UPF2 to long 3’ UTR mRNAs in the *Tdrd6*^*+/-*^ versus *Tdrd6*^*-/-*^ round spermatids showed significantly decreased binding of *Spen*, *Mdc1*, *Diap1*, *Ube2c*, *Dixdc1* and *Yap1* to UPF2 in absence of TDRD6 (Fig 7Fi–7Fvi). Thus, both UPF1 and UPF2 associations with long 3’ UTR mRNAs are affected by loss of TDRD6.

**Fig 8:**
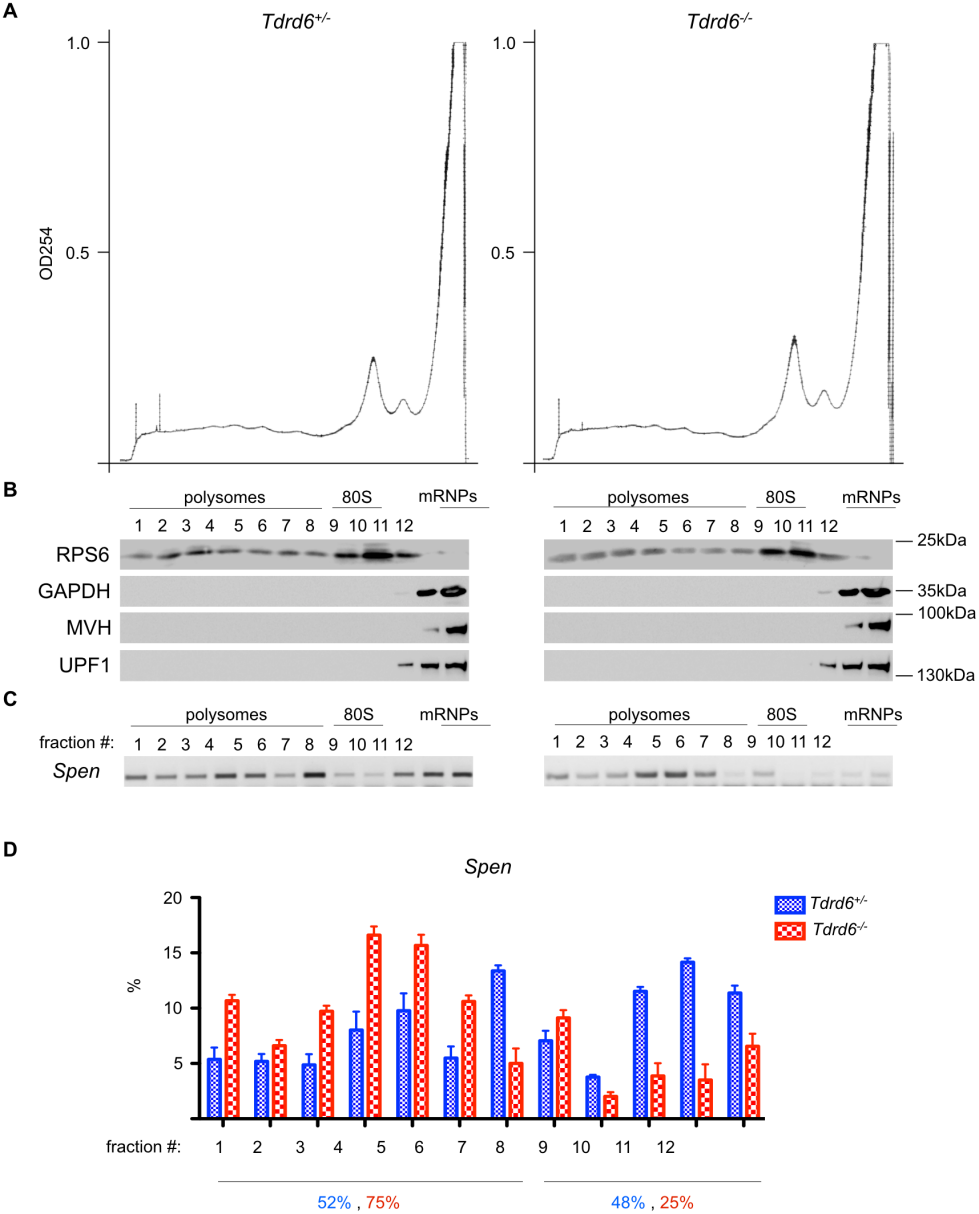
Polysome fractionation and assessment of mRNA translational potential. **(A)** Graph line showing the UV absorbance at 254nm of testis lysates from *Tdrd6*^*+/-*^ (left) and *Tdrd6*^*-/-*^ (right) mice (26 dpp) after centrifugation on a linear 15%-40% sucrose gradient. y-axis: UV absorbance in arbitrary units, x-axis: collected fractions. Representative graphs from 2 independent experiments; each sample consisted of cell preparations from 2 mice of each genotype. **(B)** Immunoblot analysis of the distribution of RPS6, GAPDH, MVH and UPF1 proteins along the fractions. **(C)** RT-PCR analysis of *Spen* mRNA distribution along the fractions. Representative image from 3 technical replicates of 2 biologically independent experiments. **(D)** Quantification of assays presented in (C). *Spen* mRNA signals in *Tdrd6*^*+/-*^ (blue bars) and *Tdrd6*^*-/-*^ (red bars) fractions were normalized to *β-actin* mRNA signals and relative abundances are presented as % of total levels.

To assess the effect of impaired UPF1-mRNA binding on the translation potential of UPF1 bound mRNAs, we performed sucrose gradient fractionation to isolate translationally active fractions, which are those rich in polysomes (fractions #1–7), translationally inactive fractions that are rich in ribosomal subunits (fractions #8–10), and ribosome-free mRNPs (fractions # 11–12) (Fig 8A). Although the majority of UPF1 protein from yeast and human cell line cultures was shown to associate with polysomes [48], we found that in 26 dpp murine testis, enriched for round spermatids, UPF1 was underrepresented in polysome/ribosome fractions #1–10, confirmed by the presence of RPS6. The majority of UPF1 was detected in fractions #11–12 containing ribosome-free mRNPs, indicated by GAPDH. The same distribution was observed for MVH. The UPF1 and MVH association with ribosome-free mRNPs was not compromised by absence of TDRD6 (Fig 8B). To test the translational capacity of UPF1-associated mRNA species we extracted RNA from each fraction and performed RT-PCR. The transcripts with long 3’ UTR, which associated less with UPF1 in *Tdrd6*^*-/-*^ samples such as *Spen* (Fig 8C and 8D), *Diap1* (S8A and S8B Fig) *Mdc1* (S8A and S8C Fig), showed relatively equal distribution in translationally active fractions #1–7 (52% for *Spen*, 43% for *Diap1* and 53% for *Mdc1*) and translationally inactive ribosome fractions #8–12 (48% for *Spen*, 57% for *Diap1* and 47% for *Mdc1*) in *Tdrd6*^*+/-*^ *samples*. In contrast, in *Tdrd6*^*-/-*^ samples, *Spen* (Fig 8C and 8D), *Diap1* (S8A and S8B Fig) and *Mdc1* (S8A and S8C Fig) showed increased abundance in the translationally active fractions #1–7 (75% for *Spen*, 68% for *Diap1* and 59% for *Mdc1*) and decreased abundance in translationally inactive ribosome and ribosome-free fractions #8–12 (25% for *Spen*, 32% for *Diap1* and 41% for *Mdc1*). Other transcripts with long 3’-UTRs, i.e *Twsg1* (S8A and S8D Fig) and *Yap1* (S8A and S8E Fig), are underrepresented in translationally active fractions #1–7 (37% for *Twsg1* and 38% for *Yap1*) and overrepresented in translationally inactive fractions #8–12 (63% for *Twsg1* and 62% for *Yap1*) in *Tdrd6*^*+/-*^ *samples*. On the other hand, in *Tdrd6*^*-/-*^ samples, *Twsg1* (S8A and S8D Fig) and *Yap1* (S8A and S8E Fig) mRNAs are more abundant in the translationally active fractions #1–7 (49% for *Twsg1* and 48% for *Yap1*) and almost equals the abundance in translationally inactive fractions #8–12 (51% for *Twsg1* and 52% for *Yap1*). Together this shows that mRNAs with reduced UPF1 binding in *Tdrd6*^*-/-*^ spermatids such as *Spen*, *Diap1*, *Mdc1*, *Twsg1* and *Yap1* associated to a larger extent with polysomal fractions compared to the controls, suggesting these mRNAs were more actively translated in *Tdrd6*^*-/-*^ spermatids.

On the other hand, mRNAs which showed no difference in UPF1 binding between *Tdrd6*^*+/-*^ and *Tdrd6*^*-/-*^ mice, such as *Ecsit* (S7Ei and S7Eii Fig), *Prss51* (S7Ei and S7Eii Fig) and *Wnt3* (S7Ei and S7Eii Fig) displayed a similar distribution pattern across all fractions in *Tdrd6*^*+/-*^ and *Tdrd6*^*-/-*^ samples (*Ecsit*: 45% in translationally active fractions #1–7 and 55% in translationally inactive fractions #8–12 in *Tdrd6*^*+/-*^ and 52% in translationally active fractions #1–7 and 48% in translationally inactive fractions #8–12 in *Tdrd6*^*-/-*^; *Prss51*: 47% in translationally active fractions #1–7 and 53% in translationally inactive fractions #8–12 in *Tdrd6*^*+/-*^ and 41% in translationally active fractions #1–7 and 59% in translationally inactive fractions #8–12 in *Tdrd6*^*-/-*^ *Wnt3*: 43% in translationally active fractions #1–7 and 57% in translationally inactive fractions #8–12 in *Tdrd6*^*+/-*^ and 35% in translationally active fractions #1–7 and 65% in translationally inactive fractions #8–12 in *Tdrd6*^*-/-*^). Overall, this data set shows that decreased binding of mRNA to UPF1 in *Tdrd6*^*-/-*^ round spermatids correlates with increased mRNA stability and translational potential.

## Discussion

The aim of the present study was to define the role of a germ cell-specific protein, TDRD6, which in spermatids resides in the CB and is a main structural component of this cell organelle whose functions remained hitherto largely unknown. CBs are considered large RNP complexes in the cytoplasm close to the nuclei of round spermatids. CBs were proposed to be sites of accumulation of mRNPs exported from the nuclei [49]. It was postulated that these mRNPs are translationally repressed through piRNAs or miRNAs or by translational regulators such as Nanos, Pum and Gemin3 [49,50]. These mRNPs would be stored or targeted to other cytoplasmic sites [51]. The dominance of Tudor domain proteins in the CB and their interactions with PIWI and other proteins was suggested to provide the molecular scaffold for CB [18]. TDRD6 and TDRD7 were shown to be indispensable for CB architecture [20,23], and in our study we used TDRD6 deficient mice. The CB has been implicated in piRNA biogenesis and retrotransposon silencing [19]. The loss of TDRD6 results in male infertility and disruption of CB architecture of which a remnant “ghost body” is left. Genome methylation remains normal as did retrotransposon silencing, which depends on MIWI and MILI, suggesting that the proper architecture of the CB is required for other functions.

To decipher some of these functions, we used proteome analysis to determine differences between *Tdrd6*^*+/-*^ and *Tdrd6*^*-/-*^ CB compositions and determined distinct perturbations of the CB proteome in TDRD6-deficient samples. In CB preparations we identified 158 proteins that depend on TDRD6 for their enrichment in CBs. To compare these with the transcriptomics data, the 158 IPI protein IDs were converted to 139 Ensemble transcript IDs (88%). Next we looked at the expression values of these transcripts in our RNA deep sequencing analysis of the *Tdrd6*^*+/-*^ and *Tdrd6*^*-/-*^ round spermatid transcriptomes. Out of the total of 139 transcripts, 96 remained unchanged between the genotypes (70%). 27 transcripts expressed lower in the *Tdrd6*^*-/-*^ round spermatids (19%) and 16 transcripts expressed higher in the *Tdrd6*^*-/-*^ round spermatids (11%). The failure to identify the 24 transcripts, that expressed lower in *Tdrd6*^*-/-*^ round spermatids, in the *Tdrd6*^*-/-*^ CB may be due to the very low expression levels. However, the the vast majority of the proteins (82%) are normally or higher expressed in *Tdrd6*^*-/-*^ round spermatids, so the failure to identify them in *Tdrd6*^*-/-*^ CB is likely a consequence of CB distortion in this mutant.

Many of the proteins absent in TDRD6-deficient CBs bear RNA binding domains pointing to a critical role of CBs in RNA metabolism, i.e. post transcriptional regulation.

Among the RNA-related proteins found within the CB proteome were components of the RNA degrading machinery, which we further investigated. Our data are in agreement with a recent study [17] that provided first insights into the molecular composition of CBs. A major subset of proteins that localizes to CBs are those implicated in RNA degradation processes e.g. mRNA decapping enzyme DCP1a and RNA endonuclease SMG6. Indeed CBs share common features with P-bodies and the NMD core factor UPF1 was found in both structures [14,38]. Here we demonstrate that key NMD factors UPF1 and UPF2 are highly expressed in post-meiotic male germ cells and accumulate in CBs implying a key role of NMD for the completion of spermatogenesis. We provide evidence that the localization of UPF1 to CBs depends on a TDRD6-supported CB structure, while UPF2 is targeted to CBs via different mechanism(s). We analyzed protein-protein interactions in the presence and absence of TDRD6. In the wild-type situation, MVH and UPF1 associated with UPF2 and TDRD6. In absence of TDRD6, MVH and UPF2 interacted with each other localizing to the CB “ghost body”, but UPF1 failed to associate with them. Thus, TDRD6 supports the formation of UPF1-containing mRNPs in the CBs.

It is very unlikely that TDRD6 itself binds directly to RNA, since there are no RNA binding domains identified in this protein. TUDOR domains bind to methylated arginines or lysines [18]. One may speculate that such methylated residues in UPF1 would enable UPF1-TDRD6 interaction, which is subject of future investigations. In any case, TDRD6 likely provides a protein scaffold, where RNA binding proteins are brought into proximity so that correctly assembled mRNPs can be formed and stabilized.

Accumulation of UPF proteins in CBs indicated that CBs support NMD, for example CBs may serve as storage sites for NMD proteins or even as sites of active NMD. The loss of TDRD6 and subsequent perturbation of UPF1 interactions did not affect the levels of PTC containing transcripts, thus did not affect PTC induced, downstream exon-exon junction dependent NMD. In *Tdrd6*^*-/-*^ round spermatids dEJ-triggered NMD is functional despite the compromised interaction of UPF1 and UPF2, suggesting an alternative pathway of UPF1 activation on a PTC containing transcript. However, we observed increased levels of transcripts with long 3’ UTR in *Tdrd6*^*-/-*^ sample, suggesting that TDRD6 supports the long 3’ UTR triggered pathway of NMD. To our knowledge, this is the first mutant that discriminates between different modes of stimulating NMD. We also demonstrate that specific mRNAs with long 3‘ UTR associate with UPF1 and UPF2 *in vivo* in round spermatids, but this association is much reduced in *Tdrd6*^*-/-*^ cells. The reduced association with UPF1 correlated with increased levels of these mRNAs and their increased translational potential in the *Tdrd6*^*-/-*^ background. The presence of a few mRNAs with either long or short 3‘ UTR that bind to UPF1 in a TDRD6-independent manner but are nevertheless altered in levels in absence of TDRD6 suggests that TDRD6 regulates the levels of some mRNAs independently of UPF1 through a distinct pathway. It has been shown that the average 3‘ UTR length of transcripts required for spermiogenesis is shorter compared to transcripts required for pre-meiotic, meiotic or testicular cell development [8]. Transcripts with shorter 3’ UTR may be more stably stored for longer periods and thus may be particularly competent for efficient translation during the last stages of spermiogenesis.

NMD is important for many developmental processes as systemic depletion of the murine *Upf1* gene results in complete loss of NMD and leads to post implantation embryonic death [52]. NMD is essential for hematopoietic stem cells and for B and T lymphocyte maturation, since conditional ablation of murine UPF2 in the hematopoietic system is detrimental to proliferation of progenitor cells and leads to up regulation of aberrant TCR and Ig locus recombination products [53]. On the other hand, NMD activity is down-regulated in neural stem cell upon neurogenic signaling to allow differentiation [54]. Thus, tissue- and cell-type specific roles of NMD exist, but are known in only a few instances. We provide the first evidence of NMD functioning in the regulation of transcripts during spermiogenesis. Successful completion of the spermiogenic program depends strongly on post-transcriptional regulation as the transcriptional production of RNA ceases from the mid to later stages because of the extensive nuclear compaction.

## Materials and Methods

### Ethics statement

The use of mice was approved by the State of Saxony animal welfare officials, Az DD24-5131/339/6 and was performed according to the national and EU guidelines.

### Animals, cells, and tissue samples

Construction of TDRD6‐deficient mice was described previously [23]. In all experiments, except otherwise noted, testes from postnatal day 26 (P26) *Tdrd6*^*+/-*^ and *Tdrd6*^*-/-*^ mice were dissected to be enriched in round spermatid cells. *Tdrd6*^*+/-*^ mice used as control for the experiments do not exhibit any phenotype and provide the targeting vector with the hCD4 gene in frame with the Tdrd6 5′ UTR and ATG (start) codon, that allows isolation of TDRD6 expressing cells through an anti hCD4-MACS approach [23]. For cell preparations enriched in round spermatids, the Tunica albuginea was removed and seminiferous tubules resuspended in 10 ml PBS and passed subsequently through 100μm and 40μm stainers. Cells were washed once with PBS and hCD4-positive cells were magnetically labeled with CD4MicroBeads (Miltenyi Biotec) and MACS isolated (Miltenyi Biotec) according to manufacturer instructions.

Testes for immunostaining were fixed in freshly prepared 4% PFA for 1h on ice, briefly washed with PBS, and incubated O/N in 30% sucrose. Testes were embedded in OCT blocks, frozen on dry ice, and cryo-sectioned at 7 μm thickness.

### Chromatoid body isolation and mass spectrometry

CBs were isolated according to Meikar et al. (2010) with some modifications. hCD4 positive cells from *Tdrd6*^*+/-*^ and *Tdrd6*^*-/-*^ adult mice were fixed in 1% PFA (Sigma) solution for 10 min at RT. The reaction was stopped by adding glycine (Roth) pH 7 to a final concentration of 0.25 M. The fixed cells were lysed by sonication in 0.5 mL of RIPA buffer (50 mM Tris-HCl at pH 7.4 (Roth), 150 mM NaCl (Roth), 1% NP-40 (Sigma), 0.5% sodium deoxycholate (Sigma), 0.1% SDS (Roth), 1 mM EDTA (Sigma), 1 mM DTT (Roth), 5mM NaF (Sigma), 1mM Na_2_VO_3_ (Sigma), 1mM PMSF (Sigma), 1x protease inhibitor cocktail complete mini (Roche)) supplemented with 100U RNAse inhibitor (Invitrogen). The lysate was centrifuged at 300g for 10 min and the CB enriched pellet resuspended in 0.5 mL of RIPA buffer. The CBs were immunoprecipitated using Dynabead Protein G (Invitrogen) coupled to rabbit polyclonal anti-MVH (Abcam) O/N at 4°C. Dynabeads were washed 4 times with RIPA buffer and the crosslinks of the isolated CBs were reversed by incubation at 70°C for 45 min in 1x Laemmli buffer.

CB samples were separated in mini-protean TGX pre-cast gradient gels (BioRad) and stained with SimplyBlue SafeStain (Life Technologies). Gel pieces were excised from the sample lanes, followed by in-gel digestion with trypsin (Promega) and extraction of the peptides. The peptides were analyzed using LC-MS/MS with an Ultimate 3000 (Dionex Corp, Sunnyvale CA) nanoLC system connected to a LTQ Orbitrap mass-spectrometer (ThermoScientific Corp., San Jose CA) equipped with an automated nanoelectrospray ion source TriVersa (Advion BioSciences, Ithaca NJ). All MS/MS samples were analyzed using Mascot (Matrix Science, London, UK; version 2.2.04). Mascot was set up to search the ipi.MOUSE_V3.76_20110304 database assuming the digestion enzyme trypsin. Mascot was searched with a fragment ion mass tolerance of 0.50 Da and a parent ion tolerance of 5.0 PPM. Oxidation of methionine and propionamide of cysteine were specified in Mascot as variable modifications. Scaffold (version Scaffold_3.6.4, Proteome Software Inc., Portland, OR) was used to validate MS/MS based peptide and protein identifications. Peptide identifications were accepted if they could be established at greater than 95% probability as specified by the Peptide Prophet algorithm [55]. Protein identifications were accepted if they could be established at greater than 99.0% probability and contained at least 2 identified peptides. Protein probabilities were assigned by the Protein Prophet algorithm [56].

### MS data analysis

Scaffold normalizes the MS/MS data between samples. Normalization is done on the MS sample level, which is the total sample run through the mass spectrometer. The normalization method that Scaffold uses is to sum the “Unweighted Spectrum Counts” for each MS sample. For the purposes of protein identification, Scaffold uses a ProteinProphet model, assigning the peptide exclusively to the protein with the most evidence. The result is that the peptide has a weight of 1 in one protein and a weight of zero in all other proteins. However, if there are two proteins, and each protein has the same peptide, then each spectrum for this peptide has ions contributed from both proteins. The “Unweighted Spectrum Count” option on Scaffold's Samples page will count this spectrum twice, once in the first protein and once in the second protein. This count is “unweighted” in the sense that the spectrum counts the same in each of the shared proteins. Scaffold counts unweighted spectra for determining protein abundance. These sums are then scaled so that they are all the same. The scaling factor for each sample is then applied to each protein group and adjusts its “Unweighted Spectrum Count” to a normalized “Quantitative Value”.

International Protein Index (IPI) accession numbers of proteins identified more than 2 fold enriched in *Tdrd6*^*+/-*^ CB (S3 Table) were uploaded to DAVID platform [26], functional annotation for protein domains from PFAM database was performed with threshold count 3 and threshold EASE 0.1. The same list was uploaded to QIAGEN’s Ingenuity Pathway Analysis (IPA, QIAGEN Redwood City) and functional analysis was performed with custom parameters.

### Immunofluorescence

Immunofluorescence labeling of frozen sections of mouse testis was performed using rabbit polyclonal anti UPF1, rabbit polyclonal anti UPF2 [35], rabbit polyclonal anti MVH (Abcam), mouse monoclonal anti SYCP3 [57], guinea pig polyclonal anti C-term TDRD6 (this study). Sections were fixed using 4% PFA for 20 minutes, blocked and permeabilized with 2% BSA, 0.1% Triton-X100 in PBS and incubated overnight with primary antibodies. Slides were washed with PBST and probed for 2 h with secondary antibodies Alexa-566-labeled goat anti guinea pig, Alexa-488-labeled goat anti rabbit, or Alexa-647-goat anti mouse (Molecular probes, Invitrogen). For double immunostaining rabbit polyclonal antibodies were labeled using the Zenon Rabbit IgG Labeling Kits (Molecular Probes, Invitrogen). Slides washed again with PBST and nuclei were visualized with DAPI. Images acquired with a Zeiss LSM 510 confocal microscope and quantification of signal intensity was done with ImageJ.

### Protein extracts, immunobloting, and immunoprecipitation

*Tdrd6*^*+/-*^ and *Tdrd6*^*-/-*^ round spermatid cell suspension fixed in 1% PFA solution for 10 min at RT to capture RNA-protein and protein-protein interactions. Cells were lysed in 0.5 mL RIPA buffer supplemented with 100U RNAse inhibitor (Invitrogen) for 20 min on ice. The lysate was centrifuged 1000 rpm for 10’ at 4°C and the protein concentration of the supernatant was quantified. 150 μg of protein extract diluted in IP buffer (50 mM Tris-HCl at pH 7.4 (Roth), 150 mM NaCl (Roth), 0.25% Triton-X100 (Sigma), 1 mM EDTA (Sigma), 5mM NaF (Sigma), 1mM Na2VO3 (Sigma), 1mM PMSF (Sigma), 1x protease inhibitor cocktail complete mini (Roche)) to a final volume of 250 μL and antibodies coupled to Dynabeads used for immunoprecipitation: goat polyclonal anti UPF1 (Bethyl), rabbit polyclonal anti MVH (Abcam), rabbit polyclonal anti TDRD6 (Antibody Verify), rabbit polyclonal anti UPF2 [35], goat IgG (Invitrogen) and rabbit IgG (Invitrogen). The beads were then washed 4 times with IP buffer. The retained proteins were resolved by SDS-PAGE and immunoblotted with with the aforementioned antibodies and mouse monoclonal anti Vinculin (Sigma).

### RNA sequencing

3 μg of total RNA per sample were used for library preparation. Ribosomal RNA was depleted by using the GeneRead rRNA Depletion Kit (Qiagen). RNA fragmentation, cDNA synthesis and further RNA-Seq library preparation was done with the NEBNext Ultra Directional RNA Library Prep Kit (New England Biolabs). After enrichment and XP bead (Agencourt AMPure Kit; Beckman Coulter, Inc.) purification, quality control was done using Fragment Analyzer (Advanced Analytical). The bar-coded libraries were equimolarly pooled and subjected to 75 bp single-end sequencing on Illumina HiSeq 2000, resulting in an average of 33 million reads per sample. Sequencing raw data were deposited in GEO database under the GSE63948 accession number. The “Tuxedo Suite” of Bowtie, TopHat, Cufflinks and Cuffdiff [39,40,58,59] was used for the alignment and expression analysis. We aligned the samples separately to the mm9 genome using the splice junction mapper Tophat (version 2.0.9), which used Bowtie 2 (version 2.1.0) for mapping. The Ensembl version 67 [41] was used as a support for the annotation during the alignment.

### RNA isolation, reverse transcription, RT-PCR and RT-qPCR

Total RNA was extracted using the TRIZOL Reagent (Invitrogen), according to the manufacturer’s instructions. The concentration and purity of the RNA samples were determined using spectrophotometer scan in the ultraviolet (UV) region. Total RNA (1 μg) was reverse transcribed (RT) with SuperScript II Reverse Transcriptase (Invitrogen) using random primer mix (NEB) according to manufacturer’s instruction. RT PCR amplification was carried out as follows with specific primers (S4 Table): 30” at 95°C, 20” at 60°C, and 30” at 72°C, for 30 cycles using DreamTaq Green DNA Polymerase (Fermentas). RT PCR products were visualized on 1% agarose gels by ethidium bromide staining. RT-qPCR amplification was carried out as follows with specific primers (S4 Table): 5” at 95°C and 30” at 60°C for 40 cycles using GoTaq qPCR Master Mix (Promega). Data analyses was performed with the ddCT method and the unpaired, one tail t-test was implemented.

### RNA immunoprecipitation

We performed anti UPF1 RNA immunoprecipitation according to [30] with modifications. Briefly, testicular cell suspension was prepared in 20 ml ice-cold PBS and subjected three times to 150 mJ/cm^2^ UV-C light (Stratagene Stratalinker 1800). After irradiation, hCD4 positive cells were selected as described above and lysed in 0.75 ml RIPA buffer supplemented with 100U RNAse inhibitor (Invitrogen) for 20 min on ice. The cell lysate was centrifuged at 13,000*g* for 10 min (4°C). The supernatant was split in 3 samples: 0.25 ml for input, 0.25 ml for anti UPF1 RIP and 0.25 ml for control IgG RIP. RIP samples were diluted with IP buffer to a final volume of 2 ml and pre-cleared with 15 μl Dynalbeads Protein G (Life Technologies). Then, 5 μL of anti UPF1 antibody (Bethyl) or normal goat IgG (Santa Cruz) were added and rotated at 4°C for 4 h. Afterwards, 15 μl Dynalbeads Protein G (Life Technologies) were added and incubated at 4°C for 1 h. After IP, the beads were washed four times with IP buffer and incubated with 1 mg/ml Proteinase K (Roth). Then, RNA extraction, RT and RT-qPCR were performed as described above. RIP RT-qPCR data analysis was performed with fold enrichment method. Briefly, each RIP RNA fractions’ CT value was normalized to the Input RNA fraction Ct value for the same RT-qPCR assay (ΔCt) to account for RNA sample specific differences as ΔCt [normalized RIP] = (Ct [RIP]—(Ct [Input]—Log_2_ (Input Dilution Factor))). Then, the normalized [RIP] fraction Ct value was adjusted to the normalized background [control Ab RIP] fraction Ct value (ΔΔCt) as ΔΔCt[RIP/control RIP] = ΔCt [normalized RIP]—ΔCt [normalized control RIP]. Fold enrichment above the sample specific control was calculated as linear conversion of ΔΔCt: Fold enrichment = 2 ^(-ΔΔCt[RIP/control RIP])^. RIP assays were conducted in 3 biological replicates and unpaired one-tailed t-test was implemented.

For UPF2 RIP, *Tdrd6*^*+/-*^ and *Tdrd6*^*-/-*^ MACS enriched round spermatid cell suspension fixed in 1% PFA solution for 10 min. After fixation, the cells were lysed in 0.45 ml RIPA buffer supplemented with 100U RNAse inhibitor (Invitrogen) for 20 min on ice, followed by 2x15 s sonication. Cell lysate was centrifuged at 1,000*g* for 10 min (4°C). The supernatant was split in 3 samples: 0.15 ml for input, 0.15 ml for anti UPF2 RIP and 0.15 ml for control IgG RIP. RIP samples were diluted with IP buffer to a final volume of 0.5 ml and 15 μl of serum containing rabbit polyclonal anti UPF2 or normal rabbit IgG (Santa Cruz) were added and rotated at 4°C for 16 h. Afterwards 30 μl Dynalbeads Protein G (Life Technologies) were added and incubated at 4°C for 2 h. After IP, the beads were washed six times with IP buffer and incubated with 1 mg/ml Proteinase K (Roth). RNA extraction, RT, RT qPCR and analysis were performed as described for UPF1 RIP.

### Sedimentation velocity centrifugation

Testicular extracts from *Tdrd6*^*+/-*^ and *Tdrd6*^*-/-*^ mice (P26) were subjected to sucrose gradient fractionation as described previously [60]. Briefly, testicular lysates (100 mM NaCl, 10 mM MgCl_2_, 20 mM HEPES, pH 7.6, 0.5% Triton X-100, 200U RNAseOUT) were centrifuged at 13,000 × g at 4°C for 2 min, and the supernatant was applied to the top of a 15–40% linear sucrose gradient. The gradient was centrifuged at 115,000 × g for 200 min (Beckman Coulter). Absorbance tracing at A254 was obtained with 759A Absorbance Detector (Applied Biosystems) and twelve fractions (1 mL) were collected manually. RNAs were extracted from 0.5 ml of each fraction using the TRIZOL Reagent (Invitrogen). Reverse transcription and RT PCR reactions performed as described above. Proteins were separated by SDS/PAGE, and Western blots were probed with rabbit polyclonal anti RPS6 (Antibody Verify) mouse monoclonal anti GAPDH (Santa Cruz), goat polyclonal anti UPF1 (Bethyl) and rabbit polyclonal anti MVH (Abcam).

### Fluorescence-activated cell sorting FACS

Cell preparations from total testis or MACS purified hCD4+ were stained with FITC-anti-Human CD4 for 20 min at 4°C and subsequently with 1 μg/ml Hoechst 33342 for 30 min at 32°C. Cells were washed with PBS and resuspended in FACS buffer (PBS, 1% BSA and 1mM EDTA). Before the analysis, 1 μg/ml PI was added to exclude dead cells. Stained cells were analyzed on a BD LSRII (BD Biosciences) using FACSDiva software (BD Biosciences). Data were analyzed using FlowJo software (TreeStar).

## Supporting Information

S1 Fig Characterization of CB proteome. **(A)** Immunofluorescence staining of *Tdrd6*^*+/-*^ and *Tdrd6*^*-/-*^ CB enriched pellet after low speed centrifugation of the cell lysate in (i) low and (ii) high magnification. The CB structures are visible as anti-MVH positive foci. DAPI (magenta) marks unlysed sperm nuclei or nuclear fragments. Scale bar 10 μm. **(B)** Immunoblotting with anti-MVH of *Tdrd6*^*+/-*^ and *Tdrd6*^*-/-*^ samples during different steps of CB purification. sup: supernatant fraction after centrifugation, pel w: wash of the CB enriched pellet fraction after centrifugation, FT: flow-through, unbound material, w1,w2,w3: subsequent washes of the Dynabeads-CB, in: input, 10% of the CB enriched pellet after the centrifugation, IP: eluted material from the Dynabeads.
(TIFF)

S2 Fig UPFs’ expression. **(A)** Reverse transcription PCR (RT-PCR) analysis of *Upf1*, *Upf2*, *Hormad1*, *Tdrd6*, *Prm2* and *Inha* mRNA in postnatal mouse testis. Lanes 0, 4, 10, 14, 18, 22, 26 and 30 represent results from wild type mice that were 0, 4, 10, 14, 18, 22, 26 and 30 days postpartum (dpp) respectively. *β-actin* is used as a loading control. **(B)** Reverse transcription quantitative PCR (RT-qPCR) analysis *Upf1* and *Upf2* mRNA expression in *Tdrd6*^*+/-*^ (blue bars) and *Tdrd6*^*-/-*^ (red bars) round spermatids. Results are presented in terms of a fold change after normalizing *Upf1* and *Upf2* mRNA levels with *β-actin* mRNA level. Each value represents the mean of three independent experiments. ns = not significant p value>0.1. **(C)** Immunoblot analysis of TDRD6, UPF1, UPF2 and MVH protein expression in total cell lysates of *Tdrd6*^*+/-*^ and *Tdrd6*^*-/-*^ round spermatids. GAPDH serves as loading control. **(D)** Isolated RNA from the flow through fraction of a-MVH IP from *Tdrd6*^*+/-*^ and *Tdrd6*^*-/-*^ samples treated with or without RNAse A was resolved in 1% agarose gel and visualized after EtBr staining.
(TIF)

S3 Fig Quantification of UPF proteins localization to CB. Bar plot showing the percentage of *Tdrd6*^*+/-*^ (blue bars) and *Tdrd6*^*-/-*^ (red bars) CBs with of without UPF1 and UPF2 signal. Images are representative from 3 independent immunofluorescence experiments.
(TIF)

S4 Fig FACS analysis of the germ cell populations enriched for round spermatids. **(A)** FACS analysis of anti-hCD4-FITC stained total testicular cell suspensions (right) and MACS-sorted germ cell suspensions (left) from *Tdrd6*^*+/-*^ and *Tdrd6*^*-/-*^ mice. **(B)** Bar-plots showing the mean fluorescence intensity of anti-hCD4-FITC stained total testicular cell suspension and MACS sorted germ cell suspension from *Tdrd6*^*+/-*^ and *Tdrd6*^*-/-*^ mice. **(C)** FACS analysis of Hoechst 33342 stained MACS sorted germ cell subpopulations from *Tdrd6*^*+/-*^ and *Tdrd6*^*-/-*^ mice. Representative plots from analyses of 2 individual mice per genotype.
(TIF)

S5 Fig Mapping statistics for transcriptomic analysis. Bar-plots showing the total number of reads (red bars) and mapped reads (blue bars) which aligned to the reference for each sample.
(TIF)

S6 Fig dEJ NMD pathways analysis. **(A)** Venn diagram shows the overlap of transcripts classified as putative dEJ NMD targets by SpliceR and Ensemble v67. Only the transcripts with sufficient reliable information are included in the diagram and further used in subsequent analysis in Fig 4A and 4B. **(B)** Schematic representation of *Auf1* pre mRNA last 3 exons (boxes) and introns (lines). **(B i-v)** Schematic representation of possible *Auf1* splicing variants. **(Bii)** and **(Biii)** splice variants are putative NMD targets. **(C)** RT-PCR analysis of *Auf1* splicing variants expression in *Tdrd6*^*+/-*^ and *Tdrd6*^*-/-*^ round spermatids using primers shown as arrows in (B). Images are representative from 2 independent experiments. **(D)** Bar chart showing the number of genes coding a single transcript (one transcript isoform with a long 3’ UTR) and the number of genes coding for multiple transcript isoforms (at least one transcript isoform with long 3’ UTR).
(TIF)

S7 Fig UPF1 binding to short 3’ UTR mRNAs. **(A)** RT-PCR analysis of short 3‘UTR mRNAs i.e. *Ecsit*, *Prss51* and long 3‘UTR mRNA *Wnt3* on UPF1-immunoprecipitated RNA from *Tdrd6*^*+/-*^ round spermatids. RNAs were immunoprecipitated from round spermatids lysate either with irrelevant goat IgG (lane 3) of anti-UPF1 (lane 4). RNA was isolated from the total lysate (lane 1) and reverse transcribed except from lane 2. **(B)** RT-qPCR analysis of *Ecsit* (i), *Prss51* (ii) and *Wnt3* (iii) in UPF1-immunoprecipitated RNA from *Tdrd6*^*+/-*^ (blue bars) and *Tdrd6*^*-/-*^ (red bars) round spermatids. Bars represent the fold enrichment (mean and standard deviation (SD) n = 3) of different mRNA species isolated by anti-UPF1 RIP over the control RIP from *Tdrd6*^*+/-*^ and *Tdrd6*^*-/-*^ round spermatids after normalization to the respective input. **(C)** RT-qPCR analysis of *Ecsit*, *Prss51* and *Wnt3* mature mRNA expression in *Tdrd6*^*+/-*^ (blue bars) and *Tdrd6*^*-/-*^ (red bars) round spermatids. Results are presented in terms of a fold change after normalizing mRNA levels with *β-actin* mRNA level. Each value represents the mean of three independent experiments. **(D)** RT-qPCR analysis of *Ecsit*, *Prss51* and *Wnt3* pre mRNA expression in *Tdrd6*^*+/-*^ (blue bars) and *Tdrd6*^*-/-*^ (red bars) round spermatids. Results are presented in terms of a fold change after normalizing mRNA levels with *β-actin* mRNA level. Each value represents the mean of three independent experiments. **(E) i)** RT-PCR analysis of *Ecsit*, *Prss51* and *Wnt3* mRNA distribution along the fractions presented in Fig 5Ei. **ii)** Quantification of assay presented in (ii). *Ecsit*, *Prss51* and *Wnt3* mRNA signals in *Tdrd6*^*+/-*^ (blue bars) and *Tdrd6*^*-/-*^ (red bars) fractions were normalized to *β-actin* mRNA signals and relative abundances are presented as % of total levels. Representative images from 2 experiments. * significant at p<0.1, ** significant at p<0.05, *** significant at p<0.01, ns not significant p value>0.1.
(TIF)

S8 Fig Distribution of mRNAs along polysome fractionation. **(A)** RT-PCR analysis of *Diap1*, *Mdc1*, *Twsg1*, *Yap1* and *β-actin* mRNA distribution along the fractions presented in Fig 5Ei. Representative images from 2 experiments. (**B-E)** Quantification of the results presented in (A). *Diap1*, *Mdc1*, *Twsg1* and *Yap1* mRNA signals in *Tdrd6*^*+/-*^ (blue bars) and *Tdrd6*^*-/-*^ (red bars) fractions were normalized to *β-actin* mRNA signals and relative abundances are presented as % of total levels.
(TIF)

S1 Table Differential expression of transcripts from *Tdrd6*^+/-^ and *Tdrd6*^-/-^ round spermatids. Column 1: Cufflinks gene ID, Column 2: Official gene name, Column 3: Chromosomal position of the gene, Column 4: Expression value in *Tdrd6*^*+/-*^, Column 5: Expression value in *Tdrd6*^*-/-*^, Column 6: log_2_ Fold change, Column 7: p-value, Column 8: Ensemble gene ID. Sheet 1: All transcripts are shown. Sheet 2: Transcripts with p-value <0.05 are shown.
(XLS)

S2 Table Differential expression of transcripts from *Tdrd6*^+/-^ and *Tdrd6*^-/-^ round spermatids grouped according to 3’ UTR length. Sheet 1: Short 3’ UTR transcripts (3‘UTR <350nt) are shown. Sheet 2: Medium 3’ UTR transcripts (350nt< 3‘UTR <1500nt) are shown. Sheet 3: Long 3’ UTR transcripts (3‘UTR >1500nt) are shown. Column 1: Cufflinks gene ID, Column 2: Official gene name, Column 3: Chromosomal position of the gene, Column 4: Expression value in *Tdrd6*^*+/-*^, Column 5: Expression value in *Tdrd6*^*-/-*^, Column 6: log_2_ Fold change, Column 7: p-value, Column 8: Ensemble gene ID.
(XLS)

S3 Table Protein components of *Tdrd6*^+/-^ and *Tdrd6*^-/-^ CBs. Column 1: Official protein name, Column 2: International Protein Index (IPI) Accession number, Column 3: Universal Protein Resource (UniProt) Accession number, Column 4: Normalized spectral counts from *Tdrd6*^*+/-*^ CB sample, Column 5: Normalized spectral counts from *Tdrd6*^*-/-*^ CB sample. Normalized spectral counts (NSC): Our protein normalizing entails averaging the unweighted spectral counts for all of the MS samples and then multiplying the spectrum counts in each sample by the average divided by the individual sample's sum. Sheet 1: NSC value of proteins >2 fold higher in *Tdrd6*^*+/-*^ CB sample. Sheet 2: NSC value of proteins >2 fold higher in *Tdrd6*^*-/-*^ CB sample. Sheet 3: NSC value of proteins that do not differ more than 2 fold between *Tdrd6*^*+/-*^ and *Tdrd6*^*-/-*^ CB sample.
(XLS)

S4 Table Primer sequences. Column 1: Primer name (including the name of the gene target), Column 2: Primer sequence, Column 3: Primer length, Column 4: Annealing temperature of the primer. Column 5: Product length of primer pairs. Columns 6–10: Primer details as previously (columns1-5) for nascent mRNA RT PCR.
(XLS)
