# Supplementary figures and images for: Chromatoid Body Protein TDRD6 Supports Long 3’ UTR Triggered Nonsense Mediated mRNA Decay
Source: PLoS Genet. 2016 May 5;12(5):e1005857. doi: 10.1371/journal.pgen.1005857 (PMC4858158; doi:10.1371/journal.pgen.1005857)

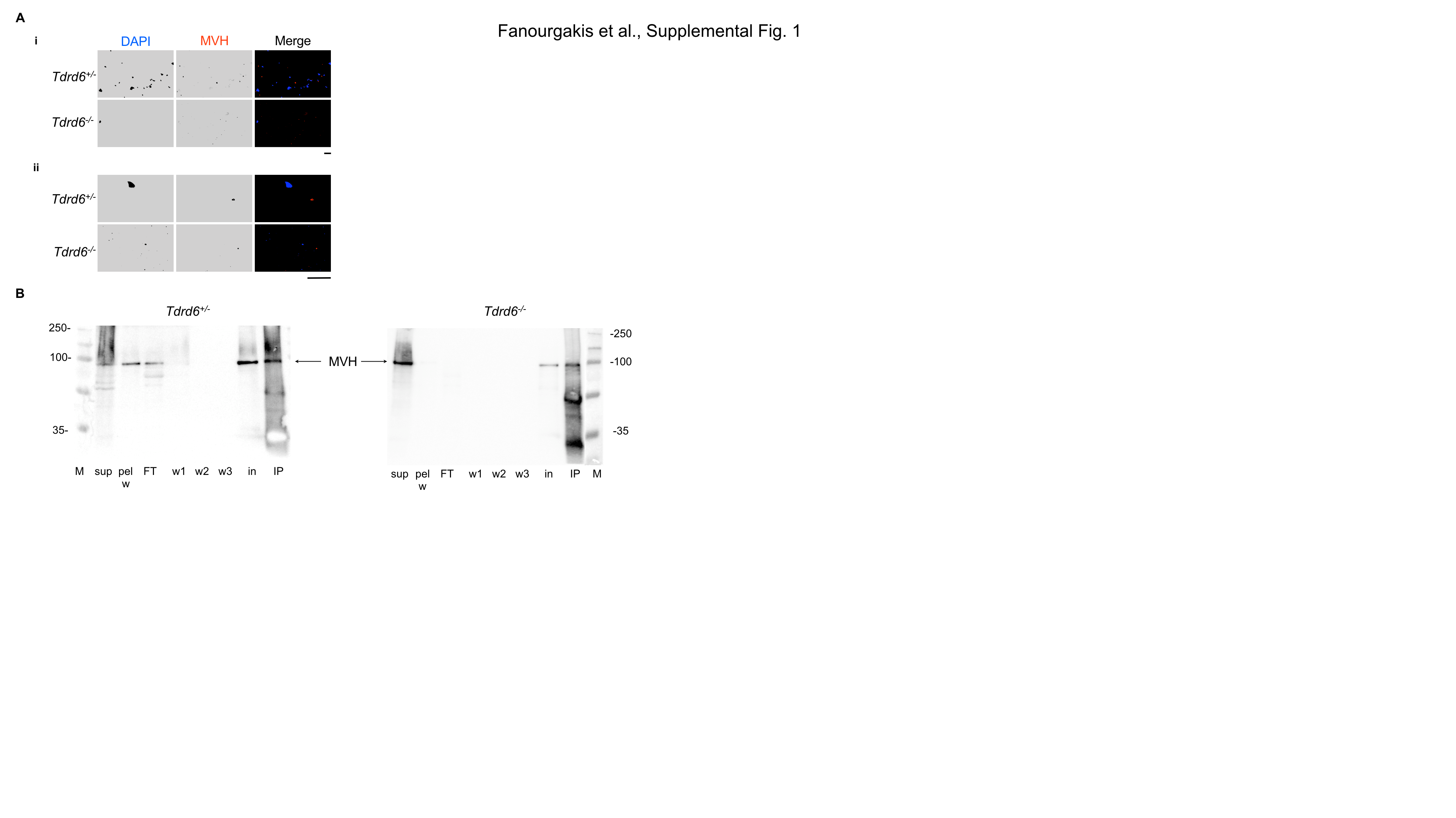

Supplement: S1 Fig — (A) Immunofluorescence staining of Tdrd6+/- and Tdrd6-/- CB enriched pellet after low speed centrifugation of the cell lysate in (i) low and (ii) high magnification. The CB structures are visible as anti-MVH positive foci. DAPI (magenta) marks unlysed sperm nuclei or nuclear fragments. Scale bar 10 μm. (B) Immunoblotting with anti-MVH of Tdrd6+/- and Tdrd6-/- samples during different steps of CB purification. sup: supernatant fraction after centrifugation, pel w: wash of the CB enriched pellet fraction after centrifugation, FT: flow-through, unbound material, w1,w2,w3: subsequent washes of the Dynabeads-CB, in: input, 10% of the CB enriched pellet after the centrifugation, IP: eluted material from the Dynabeads. (TIFF) [file pgen.1005857.s001.tiff]

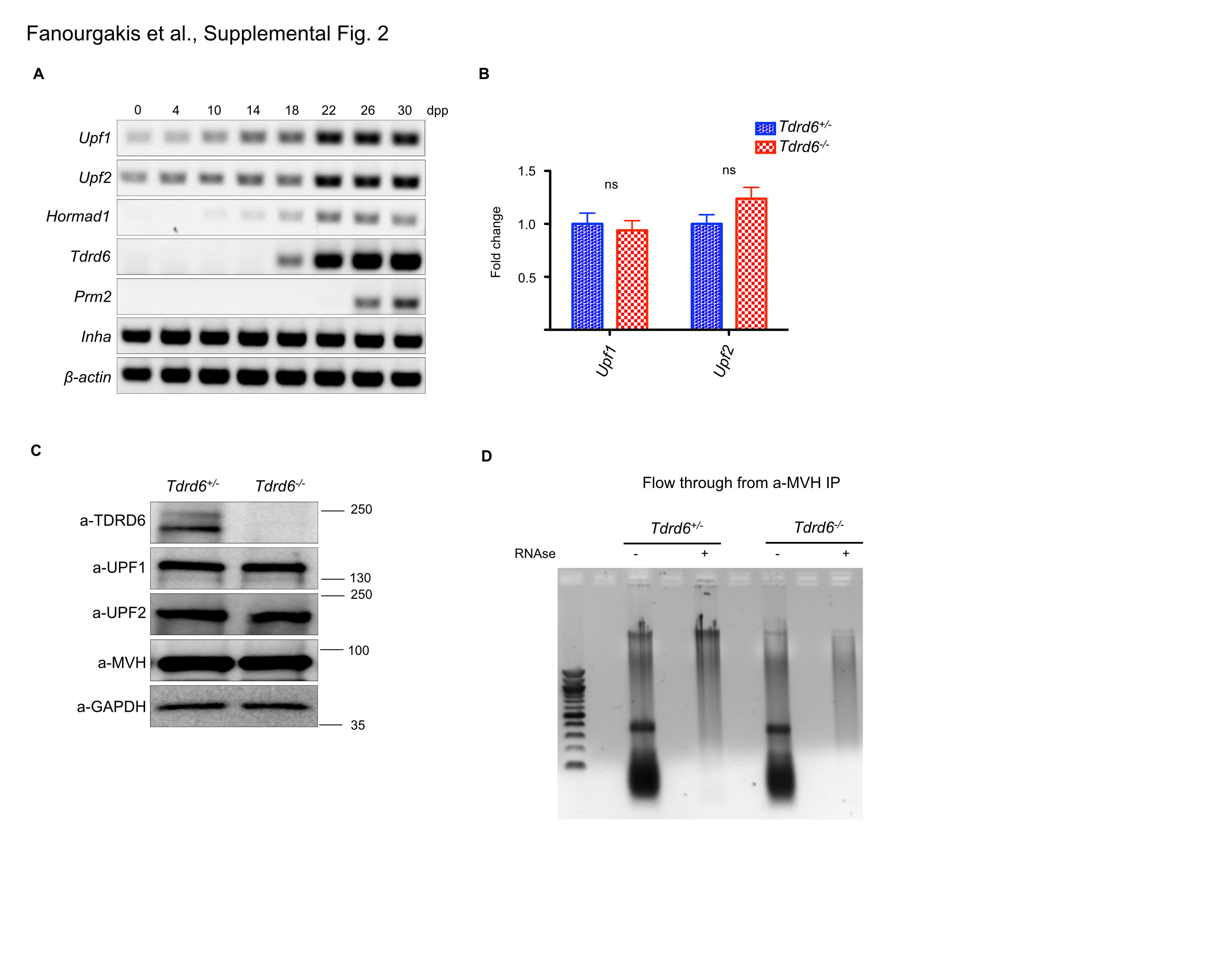

Supplement: S2 Fig — (A) Reverse transcription PCR (RT-PCR) analysis of Upf1, Upf2, Hormad1, Tdrd6, Prm2 and Inha mRNA in postnatal mouse testis. Lanes 0, 4, 10, 14, 18, 22, 26 and 30 represent results from wild type mice that were 0, 4, 10, 14, 18, 22, 26 and 30 days postpartum (dpp) respectively. β-actin is used as a loading control. (B) Reverse transcription quantitative PCR (RT-qPCR) analysis Upf1 and Upf2 mRNA expression in Tdrd6+/- (blue bars) and Tdrd6-/- (red bars) round spermatids. Results are presented in terms of a fold change after normalizing Upf1 and Upf2 mRNA levels with β-actin mRNA level. Each value represents the mean of three independent experiments. ns = not significant p value>0.1. (C) Immunoblot analysis of TDRD6, UPF1, UPF2 and MVH protein expression in total cell lysates of Tdrd6+/- and Tdrd6-/- round spermatids. GAPDH serves as loading control. (D) Isolated RNA from the flow through fraction of a-MVH IP from Tdrd6+/- and Tdrd6-/- samples treated with or without RNAse A was resolved in 1% agarose gel and visualized after EtBr staining. (TIF) [file pgen.1005857.s002.tif]

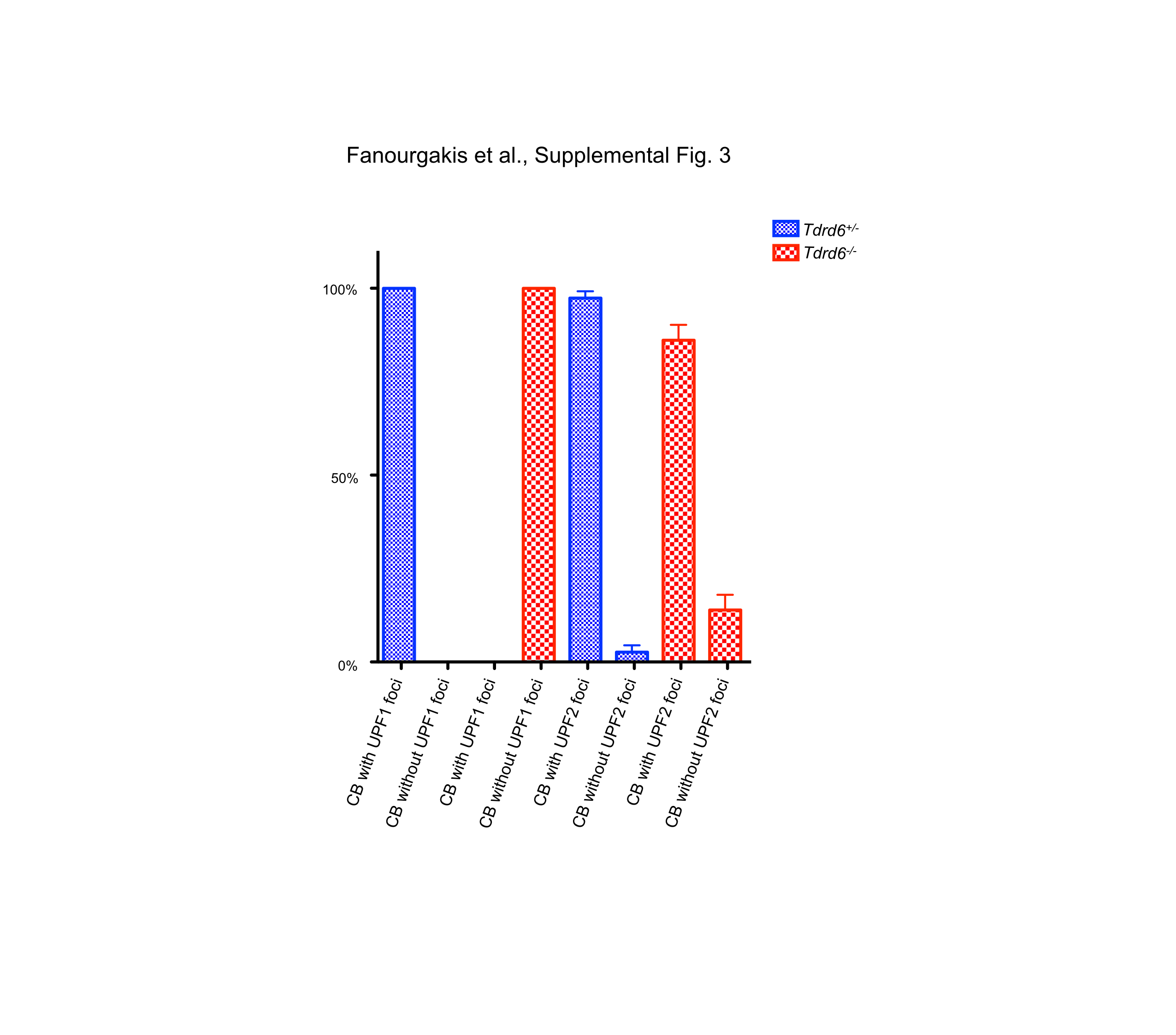

Supplement: S3 Fig — Bar plot showing the percentage of Tdrd6+/- (blue bars) and Tdrd6-/- (red bars) CBs with of without UPF1 and UPF2 signal. Images are representative from 3 independent immunofluorescence experiments. (TIF) [file pgen.1005857.s003.tif]

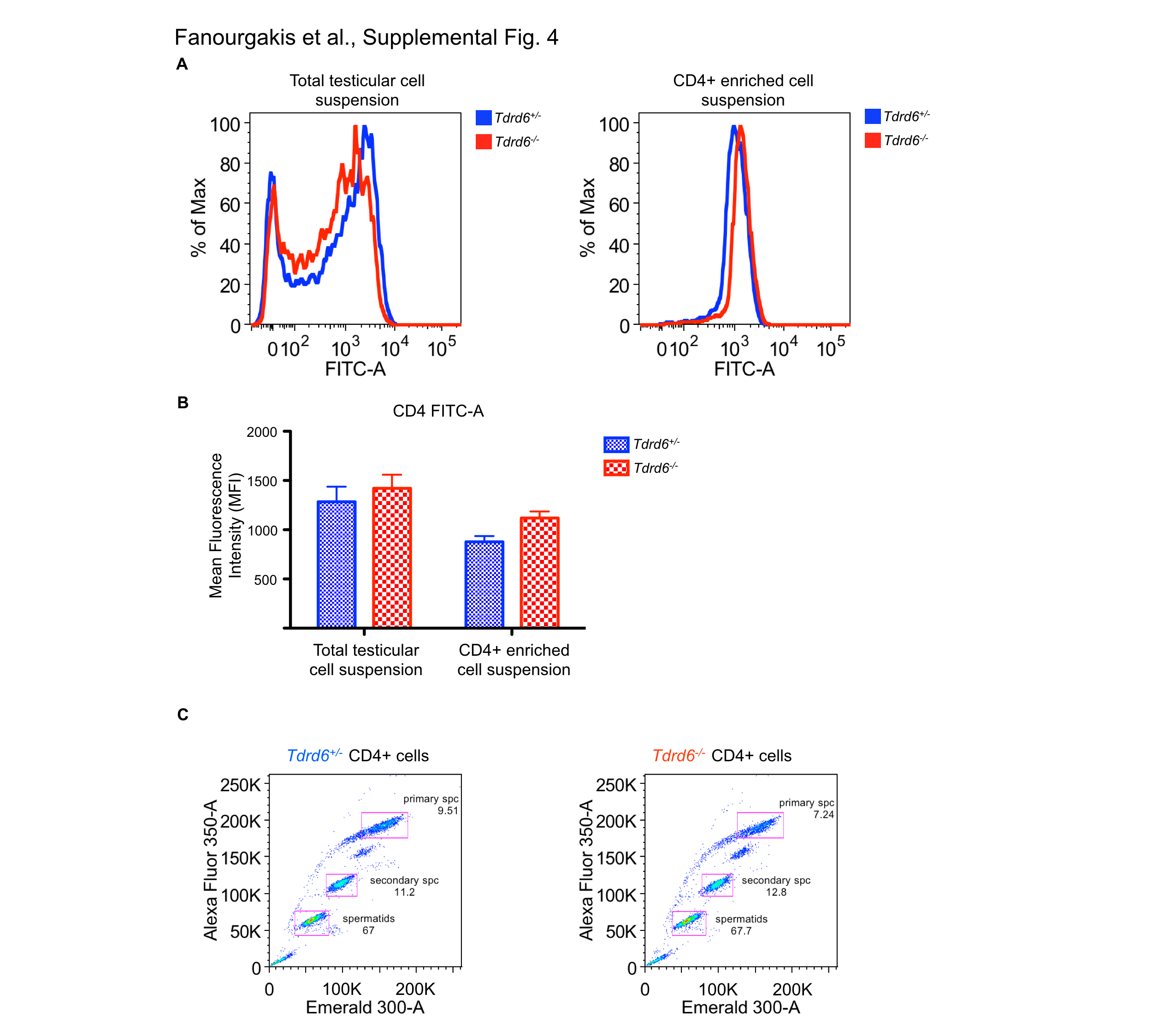

Supplement: S4 Fig — (A) FACS analysis of anti-hCD4-FITC stained total testicular cell suspensions (right) and MACS-sorted germ cell suspensions (left) from Tdrd6+/- and Tdrd6-/- mice. (B) Bar-plots showing the mean fluorescence intensity of anti-hCD4-FITC stained total testicular cell suspension and MACS sorted germ cell suspension from Tdrd6+/- and Tdrd6-/- mice. (C) FACS analysis of Hoechst 33342 stained MACS sorted germ cell subpopulations from Tdrd6+/- and Tdrd6-/- mice. Representative plots from analyses of 2 individual mice per genotype. (TIF) [file pgen.1005857.s004.tif]

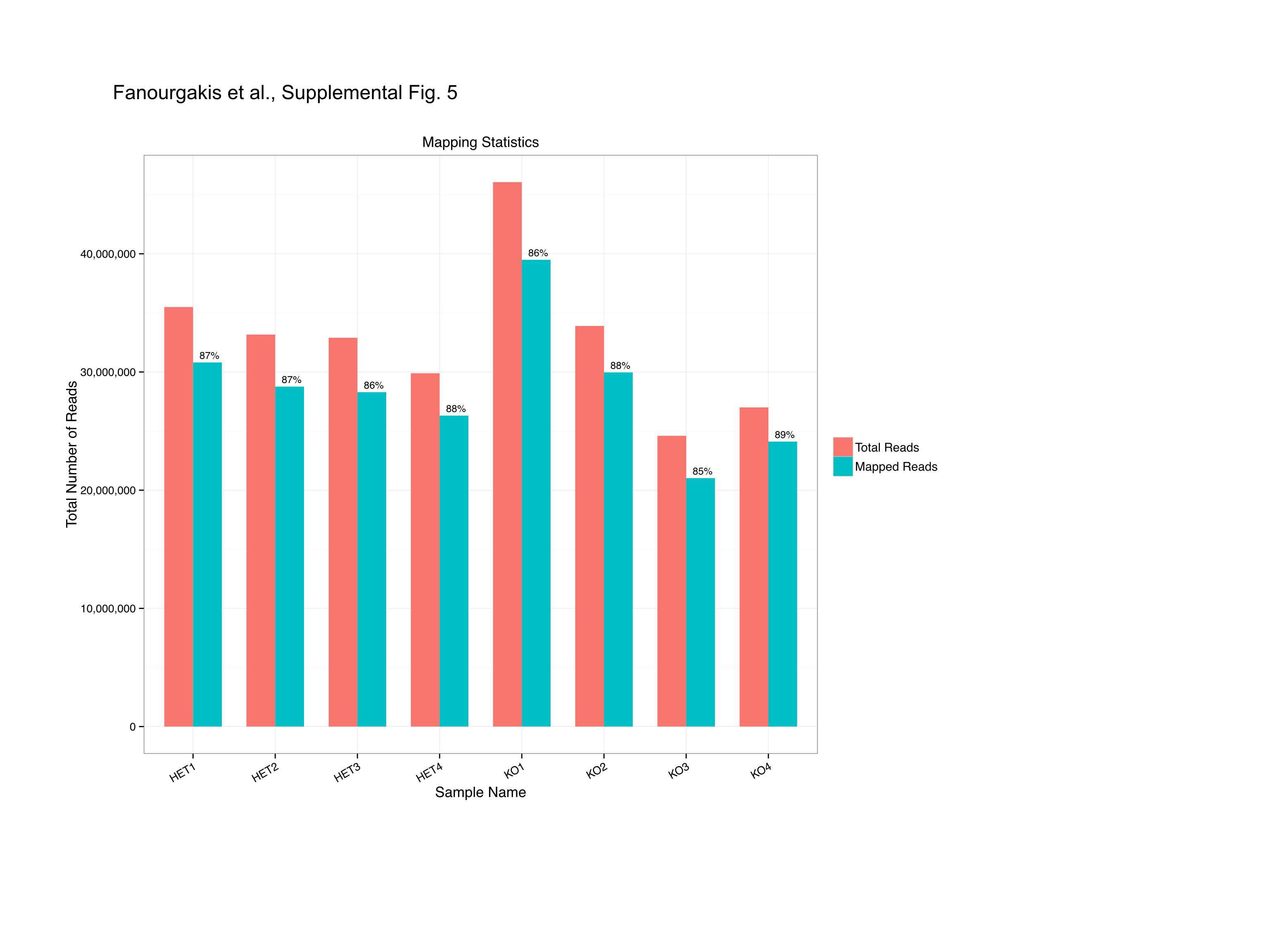

Supplement: S5 Fig — Bar-plots showing the total number of reads (red bars) and mapped reads (blue bars) which aligned to the reference for each sample. (TIF) [file pgen.1005857.s005.tif]

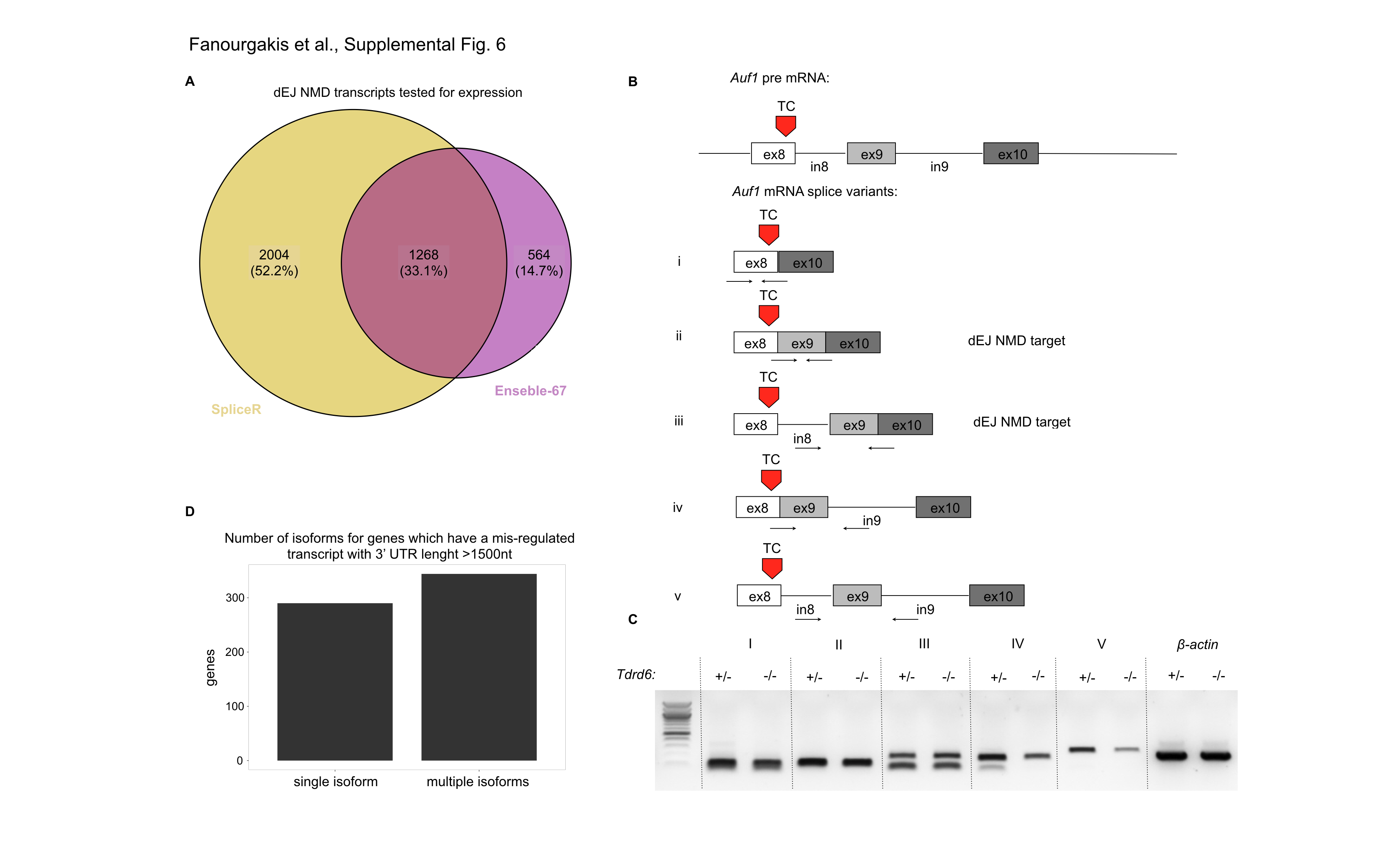

Supplement: S6 Fig — (A) Venn diagram shows the overlap of transcripts classified as putative dEJ NMD targets by SpliceR and Ensemble v67. Only the transcripts with sufficient reliable information are included in the diagram and further used in subsequent analysis in Fig 4A and 4B. (B) Schematic representation of Auf1 pre mRNA last 3 exons (boxes) and introns (lines). (B i-v) Schematic representation of possible Auf1 splicing variants. (Bii) and (Biii) splice variants are putative NMD targets. (C) RT-PCR analysis of Auf1 splicing variants expression in Tdrd6+/- and Tdrd6-/- round spermatids using primers shown as arrows in (B). Images are representative from 2 independent experiments. (D) Bar chart showing the number of genes coding a single transcript (one transcript isoform with a long 3’ UTR) and the number of genes coding for multiple transcript isoforms (at least one transcript isoform with long 3’ UTR). (TIF) [file pgen.1005857.s006.tif]

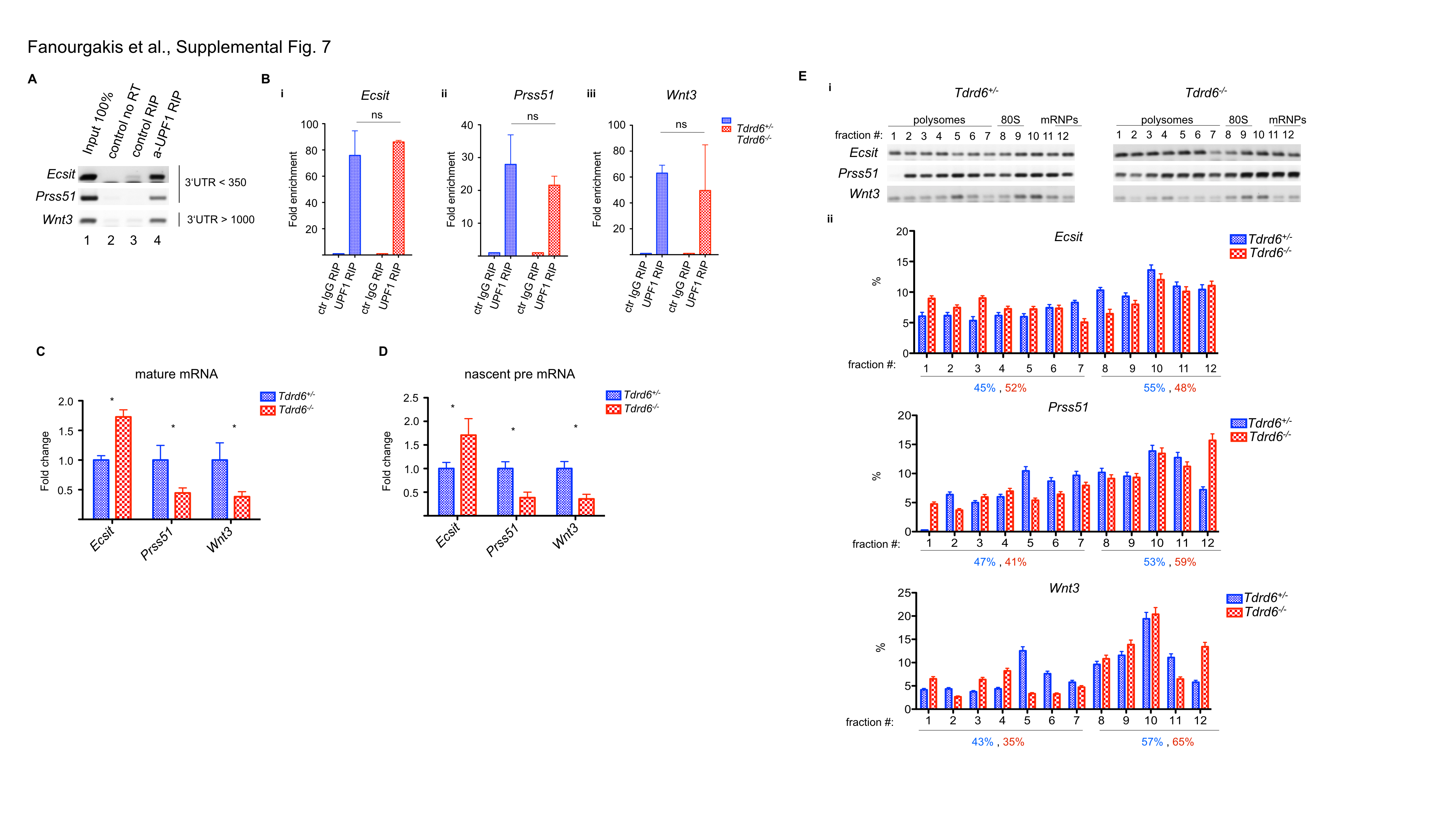

Supplement: S7 Fig — (A) RT-PCR analysis of short 3‘UTR mRNAs i.e. Ecsit, Prss51 and long 3‘UTR mRNA Wnt3 on UPF1-immunoprecipitated RNA from Tdrd6+/- round spermatids. RNAs were immunoprecipitated from round spermatids lysate either with irrelevant goat IgG (lane 3) of anti-UPF1 (lane 4). RNA was isolated from the total lysate (lane 1) and reverse transcribed except from lane 2. (B) RT-qPCR analysis of Ecsit (i), Prss51 (ii) and Wnt3 (iii) in UPF1-immunoprecipitated RNA from Tdrd6+/- (blue bars) and Tdrd6-/- (red bars) round spermatids. Bars represent the fold enrichment (mean and standard deviation (SD) n = 3) of different mRNA species isolated by anti-UPF1 RIP over the control RIP from Tdrd6+/- and Tdrd6-/- round spermatids after normalization to the respective input. (C) RT-qPCR analysis of Ecsit, Prss51 and Wnt3 mature mRNA expression in Tdrd6+/- (blue bars) and Tdrd6-/- (red bars) round spermatids. Results are presented in terms of a fold change after normalizing mRNA levels with β-actin mRNA level. Each value represents the mean of three independent experiments. (D) RT-qPCR analysis of Ecsit, Prss51 and Wnt3 pre mRNA expression in Tdrd6+/- (blue bars) and Tdrd6-/- (red bars) round spermatids. Results are presented in terms of a fold change after normalizing mRNA levels with β-actin mRNA level. Each value represents the mean of three independent experiments. (E) i) RT-PCR analysis of Ecsit, Prss51 and Wnt3 mRNA distribution along the fractions presented in Fig 5Ei. ii) Quantification of assay presented in (ii). Ecsit, Prss51 and Wnt3 mRNA signals in Tdrd6+/- (blue bars) and Tdrd6-/- (red bars) fractions were normalized to β-actin mRNA signals and relative abundances are presented as % of total levels. Representative images from 2 experiments. * significant at p<0.1, ** significant at p<0.05, *** significant at p<0.01, ns not significant p value>0.1. (TIF) [file pgen.1005857.s007.tif]

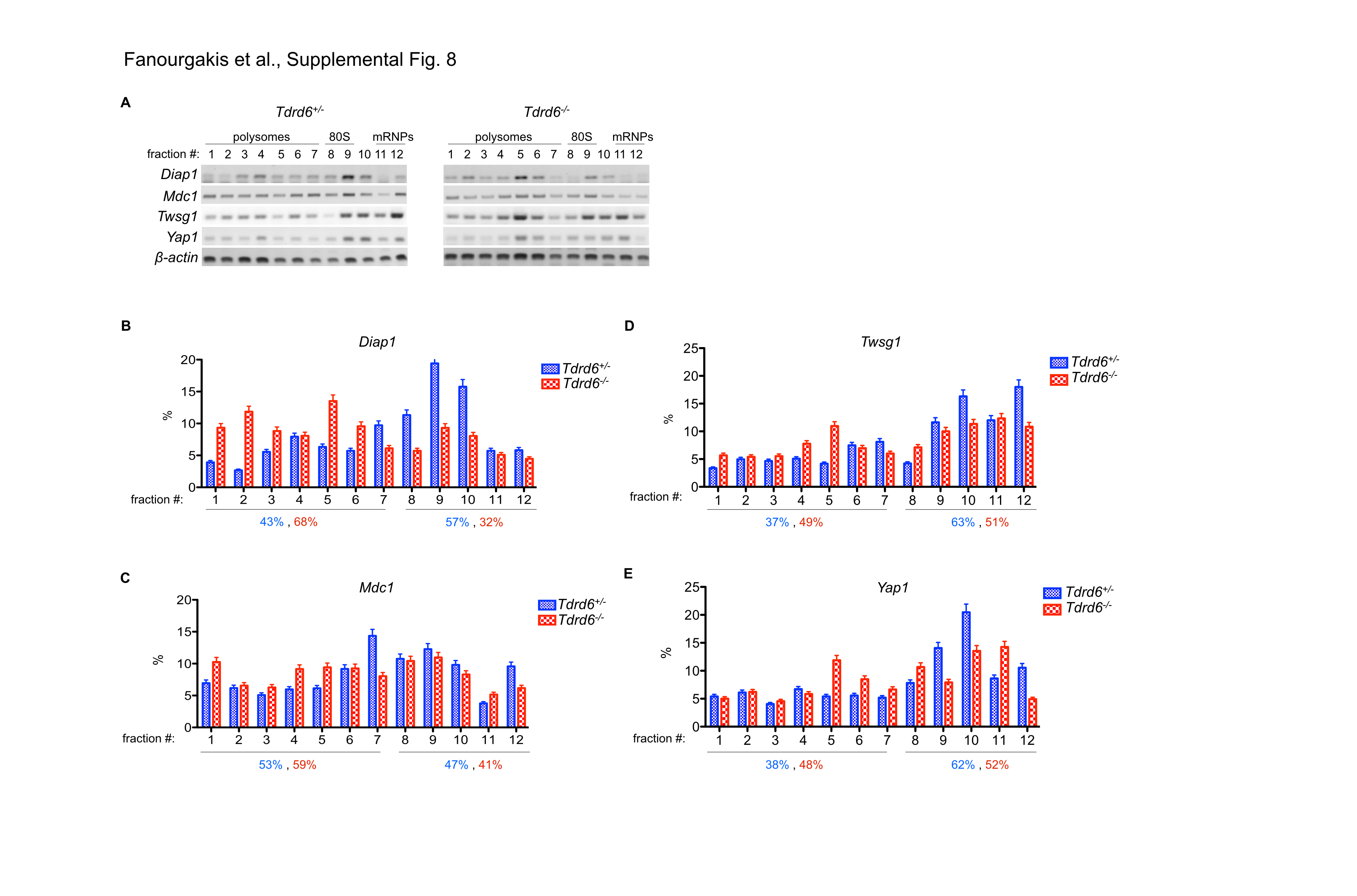

Supplement: S8 Fig — (A) RT-PCR analysis of Diap1, Mdc1, Twsg1, Yap1 and β-actin mRNA distribution along the fractions presented in Fig 5Ei. Representative images from 2 experiments. (B-E) Quantification of the results presented in (A). Diap1, Mdc1, Twsg1 and Yap1 mRNA signals in Tdrd6+/- (blue bars) and Tdrd6-/- (red bars) fractions were normalized to β-actin mRNA signals and relative abundances are presented as % of total levels. (TIF) [file pgen.1005857.s008.tif]
